# Advanced fabrication and characterization of robocasted halloysite-hydroxyapatite nanocomposite scaffolds for hard tissue regeneration

**DOI:** 10.3389/fbioe.2026.1909411

**Published:** 2026-10-21

**Authors:** A. J. Ashwin, J. M. Jafferson, Davidson Jebaseelan

**Affiliations:** 1 School of Mechanical Engineering, Vellore Institute of Technology, Chennai, India; 2 Centre for Human Movement Analytics and School of Mechanical Engineering, Vellore Institute of Technology, Chennai, India

**Keywords:** ceramic 3D printing, halloysite nanoclay, hydroxyapatite scaffold, robocast scaffold, robocasting

## Abstract

The rising frequency of bone injury and disease necessitates the need for bone replacement materials and scaffolds. Hydroxyapatite (HAP), a natural element of hard tissue, is frequently employed for bone defects treatment due to its biocompatibility and identical structure. But its brittleness limits its application in load bearing. In this present study, porous scaffolds with improved mechanical and biological performances were fabricated by combining HAP with Halloysite nanoclay (HNC 1 wt.% to 3 wt.%) using the Robocasting technique. Hydroxypropyl methylcellulose (HPMC, 1–2 wt.%) was used as the binder to prepare printable pastes. An optimal concentration of 1.5 wt% for the HPMC binder was identified from rheological measurements. The green scaffolds were printed, dried, and sintered at 1,100 °C. The microstructure and distribution of HNC within the scaffolds were characterized by FESEM. The reinforcement concentration was limited to 3wt.% due to the microcracks and agglomeration observed in the 3 wt.% scaffold. Vickers hardness increased from 245.7 H V (pure HAP) to 271.7 H V (2 wt.% HNC + HAP scaffold) with a plateau from agglomeration. Also, the porosity is observed between 38% and 40%. The biological evaluation comprised Alamar Blue proliferation, MTT cell viability (MG-63), and antimicrobial testing against *Staphylococcus aureus*. The antimicrobial assays showed comparatively limited zones of inhibition for HAP and HAP–HNC composites (max ∼8.7 mm for HAP) in comparison to ciprofloxacin (25.3 mm). All HAP–HNC scaffolds demonstrated *in vitro* cytocompatibility, wherein the addition of HNC increased cell survival and proliferation compared to the pure HAP. The 2–3 wt.% HNC demonstrated the highest biological response. Hence, the improved cytocompatibility, tolerable antibacterial behavior, and superior mechanical qualities of the HAP–HNC scaffolds make them suitable candidates for bone tissue engineering applications.

## Graphical Abstract

**Figure:**
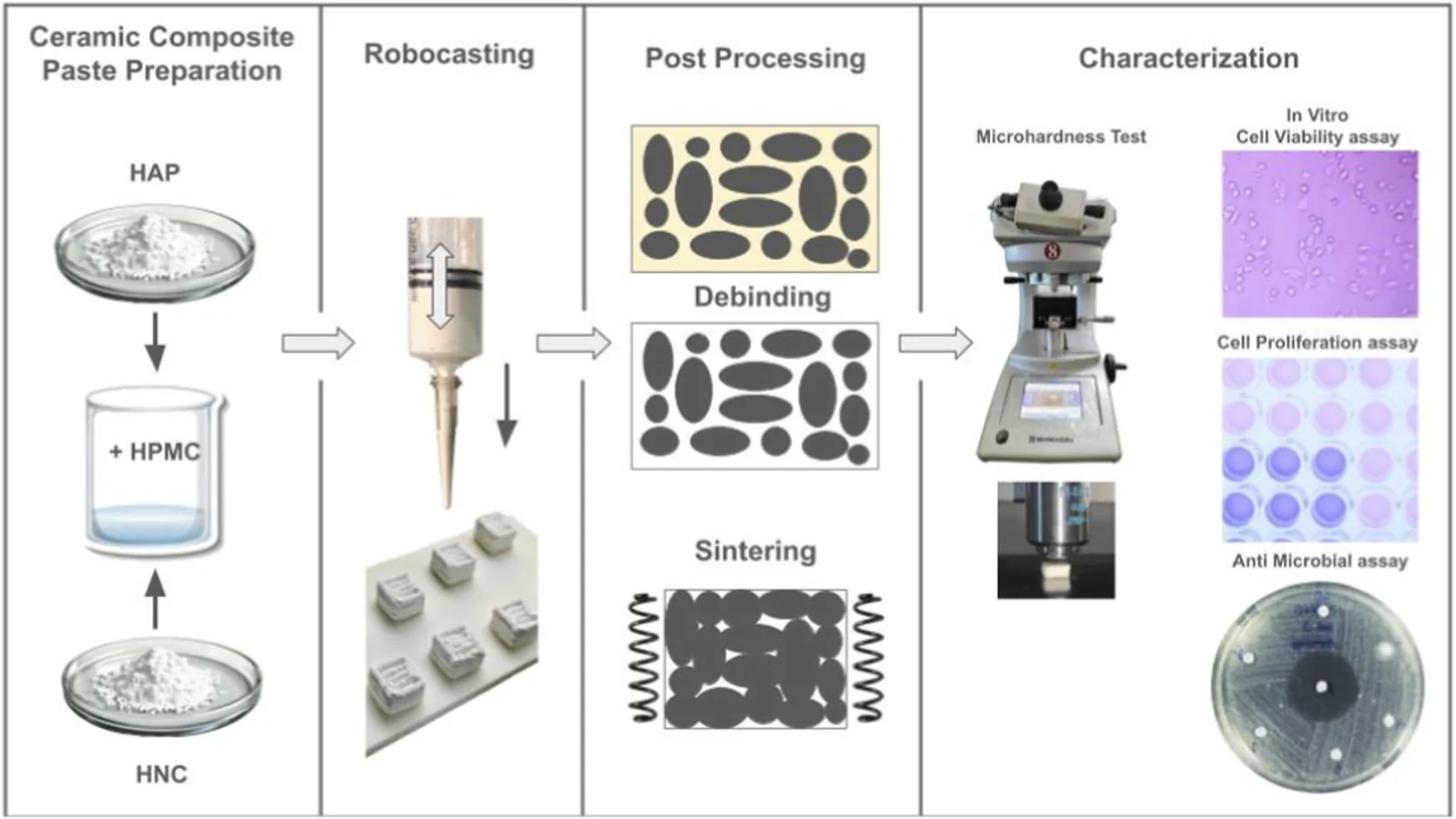

## 1 Introduction

The demand for productive bone tissue replacement materials has increased due to the rising rate of bone related injury, trauma, and orthopedics diseases. While the bone has an innate capacity to repair and regenerate on its own, its natural regenerative capacity is often insufficient for repairing critical sized defects, which necessitates clinical interventions (Babakhani et al., 2025). To treat such bone impairments allografts and autografts have been conventionally employed as bone replacements. However, they suffer from certain notable drawbacks. For instance, the traditional autograft procedure is invasive and expensive, sometimes resulting in donor site complications. Meanwhile, autografts also lose their mechanical strength and osteogenic properties when subjected to sterilization processes (Sarker et al., 2019). These disadvantages are overcome by bioceramics. Unlike industrial ceramics, they are designed to interact with the biological systems of the human body in a safe and effective manner. Bioceramics are generally divided into several categories such as calcium silicates, calcium phosphates, bioactive glass, and other glass-based ceramics. These materials are commonly employed in different clinical applications such as void fillers, bone cement, and scaffold systems (Yang and Wu, 2025). Among these Hydroxyapatite (HAP) from calcium phosphate family is recognized as a gold standard for bone reconstruction and received notable attention due to its chemical resemblance to bone mineral (Ghorbani et al., 2026). Natural bone is a hierarchical nanocomposite that consists of inorganic HAP crystals enclosed in an organic collagen matrix. This exclusive architecture gives bone the required mechanical strength, biological signaling, and fracture toughness essential for self-repair. HAP is also valued for its bioactivity, compatibility, and resorbability, which promote its strong integration with existing tissue. Nevertheless, the use of HAP is limited owing to its brittle nature (Zúquete et al., 2026). Bone scaffolds are porous and three-dimensional templates that serve as transient extracellular matrices to promote the development of new bone tissue. In addition to encouraging cellular attachment, proliferation, and differentiation, they offer structural support at the location of the bone defect (Todd et al., 2024; Verma et al., 2026). The major objective of a bone scaffold is to restore and preserve biological behavior in the damaged tissues caused by illness, aging, and injury (Jahani et al., 2020). These structures should be mechanically stable to sustain physiological loads, biocompatible, and biodegradable at a rate consistent with the formation of new bone (Yang et al., 2024; Decl et al., 2014). Scaffold architecture is the core aspect of tissue engineering and serves as a fundamental template for tissue regeneration, where its precise physical properties like pore size, geometry control and spatial distribution are the behavioral fate of the invading cells. By replicating the natural extracellular matrix (ECM), these structural cues influence important cellular signaling pathways that cause tissue-specific development (Zhao et al., 2026; Martín-Compaired et al., 2025). For instance, modern scaffold designs often incorporate a planned gradient of porosity to balance opposing biological needs. While the smaller pores promote robust initial cell attachment, bigger interconnecting channels are important for increasing vascular invasion and ensuring deep nutrition penetration, both are vital for osteogenic and angiogenic induction (Laubach et al., 2022; Shen et al., 2023). Manufacturing methods of ceramic scaffolds are broadly differentiated into conventional and modern categories. Conventional manufacturing methods include electrospinning, solvent casting, and freeze drying, whereas recent trend has focused on modern methods, especially Additive Manufacturing (AM), because of its capacity to produce accurate interior pore layouts and patient-specific geometries (Tang et al., 2025; Ingole et al., 2021). AM has become a transformative technology for producing ceramic scaffolds, especially in the field of tissue engineering and bone repair in contrast to traditional methods which are often time consuming and limited in design flexibility (Cárdenas Correa et al., 2026). Robocasting, also known as Direct ink writing, is an AM technique, which provides several research compatible advantages compared to other AM techniques like selective laser sintering (SLS) and stereolithography (SLA). Robocasting technique is particularly suited for research and development where minimal material is required, while other requires large quantity of powders or specialized resins (Ashwin et al., 2025; Ghosh et al., 2026). This feed stock efficiency makes robocasting ideal, when dealing with expensive and limited ceramics such as HAP, β-TCP, and zirconia (Ravoor and E. S, 2022). Robocasting was specially invented to fabricate ceramic products (Zhang et al., 2022), wherein a dense ceramic paste is deposited layer by layer by extruding through a fine nozzle following a Computer Aided Design (CAD) model, enabling precise control over the scaffold’s structure and pore architecture. The ceramic paste is prepared by blending the ceramic powder with a polymeric binder until a paste with an extrudable viscosity is achieved. Following the preparation of the green ceramic scaffolds, the binder is eliminated through a thermal debinding process (Zhao et al., 2025). The scaffolds are further moved to a controlled sintering process, which is most important in the fabrication of ceramic scaffolds (Sun et al., 2025). Robocasting stands out for its capacity to work with a wide variety of ceramic materials like calcium phosphates, bioactive glass, and complex composites and for its ability to produce high resolution, mechanically robust structures with customizable properties (Monfared et al., 2026). As described earlier, the brittleness and poor mechanical properties of HAP scaffolds often limit their load bearing applications (Guimarães et al., 2010). The introduction of a unique reinforcement material such as the Halloysite Nanoclay (HNC) in the HAP matrix is observed to overcome this limitation. Halloysite is a naturally occurring clay mineral and an allotrope of kaolin. It is produced by weathering and hydrothermal alteration of aluminosilicates (Farzadnia et al., 2013). HNC is an ideal candidate for medical mineralogy due to its biocompatibility, low toxicity, and environmental stability. Further, the unique tubular morphology of HNC makes it a favorable reinforcement nanomaterial in biomedical scaffolds (Shadi et al., 2026; Ianev et al., 2026). The synergistic combination of HAP’s osteoconductive nature and HNC’s reinforcement results in the development of 3D scaffolds with improved cell attachment, proliferation, and differentiation. This combination materially elevates the scaffold’s performance in hard tissue defect repair and other biomedical applications (Zohoori et al., 2024). Because of HNC’s biocompatibility and non-toxic properties, it improves the bone regeneration potential of composite scaffolds by offering high surface area and providing protein delivery or growth factor, which are key aspects in bone tissue engineering (Raja and Yun, 2016). Also, by functioning as a carrier of bioactive molecules like alkaline phosphate, HNC enhances the osteoconductivity of the scaffold. In this study HNC was incorporated in the HAP scaffold structure fabricated using Robocasting technique (Khalifa et al., 2025). The scaffolds were fabricated by mixing the HAP-HNC nanocomposite ceramic powder with Hydroxypropyl Methylcellulose (HPMC) polymer binder. HPMC is a water soluble, hydrophilic biopolymer derived from cellulose through etherification (Janmohammadi et al., 2023; Feroz and Dias, 2021). In the field of bone tissue engineering, HPMC serves as a critical binder and viscosity-enhancing agent for the HAP scaffolds, which are designed to mimic the mineral component of natural bone (Hanafy et al., 2026). During post processing, the fabricated scaffold was dried at 120 °C and sintered at 1,100 °C. Due to the thermal stability of the HNC, the reinforcement was retained within the matrix. Rheological studies were conducted for the ceramic pastes formulated by adding HAP with different concentrations of the binder to find out the proper mixture for printing. Subsequent to this, the biological characterization was performed on the fabricated HAP + HNC nanocomposite ceramic scaffolds and the fluid uptake ability of the scaffolds was evaluated by swelling study. Also, the antibacterial property of the scaffolds was evaluated by antimicrobial study. The cytocompatibility of the fabricated scaffolds were analyzed by *in vitro* cell viability assay and cell proliferation assay. Our previous work showed that the addition of HNC to HAP enhances mechanical strength and controlled degradation (Ashwin et al., 2025), physical stability is not sufficient to ensure clinical efficacy. This study bridges material structure and biology by providing the first necessary evaluation of cell viability, proliferation and antimicrobial performance for this novel 3D printed composite.

## 2 Materials and methods

### 2.1 Materials

Hydroxyapatite (HAP) Nano powder was purchased from Vedayukt India Pvt. Ltd., India, while Halloysite nanoclay (HNC) was purchased from Sigma Aldrich. Hydroxypropyl Methylcellulose was purchased from CDH India.

### 2.2 Nano composite paste preparation

The hydroxyapatite (HAP) with a stoichiometric Ca/P molar ratio of 1.67 is used as the matrix material. The HNC suspensions were prepared by dispersing about 1wt%, 2wt%, and 3wt% of the purchased Halloysite nanoclay separately in 10 mL of ethanol and sonicating using probe sonicator at 40% with 2 s ON and 2 s OFF condition for 15 min. The three set of HAP slurries was prepared by adding 20g of the HAP ceramic to 100 mL of DI water separately. Later, 10 mL of the prepared HNC suspensions were added to the HAP slurry and stirred with a magnetic stirrer for 12 h at room temperature to produce uniformly dispersed HAP–HNC nanocomposite slurry. This slurry was then placed in a hot dry oven at 120 °C for 12  h to produce a nanoceramic composite cake. The composite cake was ground using mortar and pestle to obtain a fine powder. The polymeric binder solution was prepared by dissolving HPMC in hot DI water and stirring using a magnetic stirrer (REMI) at 600 RPM till it reached room temperature. Polymeric binder solutions of 3 different concentrations (1wt%, 1.5wt%, and 2wt%) were prepared and tested to find out the proper combination of HPMC binder suitable for robocasting the HAP-HNC composite scaffolds. The HAP–HNC composite ceramic powders were mixed with the HPMC polymeric binder to form a ceramic composite paste. The extruding capacity and strand stability of the prepared ceramic composite pastes were determined by extruding them using a 0.8 mm nozzle at 50% feed rate on 3D printed multiple pillar structures spaced at 1, 2, 3, 4, 8, and 16 intervals (Therriault et al., 2007). Based on the observations, the composite slurry with 1.5wt% of HPMC binder was chosen for making scaffolds.

### 2.3 3D fabrication of nanocomposite scaffolds

For 3D fabrication, a 10 × 10 × 7 mm^3^ scaffold structure was designed using CAD software and exported as an STL file. The STL file was then imported into the slicing software (Repetier Host) and the model was sliced with 50 infill percentage and the corresponding G-code was generated after slicing. The prepared nanocomposite paste was loaded into a 10cc paste dispensing cartridge and placed in a Robocasting set up. The generated G-code was loaded to the robocasting controller and the designed scaffold structures were printed layer by layer. The printing platform made up of Alumina allowed direct transfer of the green scaffolds to the furnace. This avoids structural deformation during removal from the built platform. The printed nanocomposite green scaffolds were dried at 120 °C using a hot air oven for 12 h. The hydroxyapatite sintering temperature is restricted below 1,200 °C to avoid de-hydroxylation and thermal decomposition into secondary calcium phosphate. Also sintering at lower temperatures creates insufficient thermal energy to drive effective grain boundary and bulk volume diffusion (Ruys et al., 1995). This results in failure of ceramic particles to form solid neck interconnections and incomplete densification (Yang et al., 2008). To overcome this the scaffolds were sintered at 1,100 °C. This ensures the same temperature from the surface to core and achieve uniform strength throughout the scaffolds. The soaking time is given as 4 h in a high temperature programmable box furnace. The heat in the furnace was controlled carefully by setting the temperature ramp up rate at 2 °C/min to achieve uniform shrinkage and to avoid stress cracking, while the scaffolds were cooled down within the furnace itself.

### 2.4 Rheological characterization

The rheological characterization of the HAP ceramic paste was carried out using Anton Paar MCR 502 rheometers with crosshatch parallel assembly plate at IIT Madras, Chennai. The prepared ceramic paste with 1wt%, 1.5wt%, and 2wt% of binder concentrations were placed individually between the plate assembly, with a gap of 1 mm maintained between the top and bottom plates. The shear rate was increased from 0 to 1,000 1/s and the values were noted. The rheological properties of the prepared pastes were then evaluated.

### 2.5 Material characterization

The microstructure of the HAP and HNC reinforced HAP scaffold structures were observed using Field Emission Electron Microscopy (FESEM, Quanta FEG 250). The retention and dispersion of HNC in the HAP matrix were determined from the FESEM images. The elemental composition of the nanocomposite scaffolds was determined using Energy Dispersive X-ray spectroscopy (EDS).

### 2.6 Mechanical study

The Young’s modulus of the sintered HAP and HNC incorporated HAP scaffolds were evaluated using Shimadzu Vickers microhardness testing instrument under a constant load of 0.5 N with a 15 s dwell time. The 3D printed samples for this study were prepared by polishing the top and bottom layers. The microhardness values were calculated based on the measured lengths of the indentation diagonals using Equation 1. Three samples (n = 3) have been tested from each set. F = Load in Kgf, d = mean of the diagonals

$$vickers\;hardness\;\left(HV\right)=1.854\frac{F}{d^{2}}$$ (1)

### 2.7 Swelling study

For swelling studies, the scaffolds were dried using a vacuum oven and dried weight (W_d_) was measured. The dried scaffolds were immersed in 1x phosphate buffered saline (PBS) and incubated at 37 °C. The scaffolds were removed from the medium and the wet scaffolds weight (W_t_) were measured in time interval of 12 h s, 24 h s and 48 h s. The swelling ratio of scaffolds were evaluated using Equation 2. Three samples (n = 3) have been tested from each set.

$$Swelling\;Ratio\;\left(\%\right)=\frac{W_{t}-W_{d}}{W_{d}}\times 100$$ (2)

### 2.8 Porosity measurement

The porosity percentage of sintered HAP and HNC incorporated HAP scaffolds were measured using the liquid displacement method employing Archimedes principle (ASTM B962) (Mondal et al., 2020). The sintered scaffolds were dried using a vacuum oven and dry weight (W_d_) was measured. The dry scaffolds were soaked in absolute ethanol under vacuum for 1h. Then the soaked (W_s_) and suspended (W_a_) scaffolds are weighed. The porosity percentage of scaffolds were evaluated using Equation 3.

$$Porosity\;\left(\%\right)=\frac{W_{s}-W_{d}}{W_{s}-W_{a}}\times 100$$ (3)

### 2.9 Antimicrobial assay

The antibacterial activity was evaluated by using the Zone inhibition method (Kirby Bauer disk diffusion). The Mueller-Hinton agar (MHA) plates were inoculated by spreading them with 100 µL of the bacterial culture, *S. aureus* (inoculation was prepared by adjusting 0.5McFarland unit- Approx cell density (15 × 108 CFU/mL from) Mueller-Hinton broth) and followed by different concentrations (00–100 mg/mL). One disc in each plate was loaded with solvent alone which served as the vehicle control and the Ciprofloxacin disc (10 µg) was taken as the positive control. The plates of *Staphylococcus aureus* were incubated (Asil scientific corp. India) at 37 °C for 24 h. The clear zones created around the disc were measured and recorded.

### 2.10 Cell viability assay

The cytotoxicity of the HAP and HNC reinforced HAP scaffolds on the MG-63 (NCCS Pune) cell line was examined by MTT Assay. The cells with a density of 10000/well were cultured in 96 well plates for 24 h in the RPMI 1640 medium (RPMI 1640 Medium- AT162-1L- HIMEDIA) supplemented with 10% FBS (Fetal Bovine Serum - HIMEDIA-RM 10432) and 1% antibiotic solution (Penicillin-Streptomycin-Sigma-Aldrich P0781) at 37 °C with 5% CO_2_. On the next day, the cells were treated with different concentrations of the samples. Stock solutions of the samples were prepared in dimethyl sulfoxide (DMSO), which were further diluted to get different concentrations in incomplete Cell culture Medium (Without FBS). After incubation for 24 h, the MTT solution (5 mg/mL) was added to the cell culture, which was further incubated for 2 h. Cells without treatment were considered as control and cells without MTT were considered as blank. At the end of the experiment, the culture supernatant was removed and the cell layer matrix was dissolved in 100 µL dimethyl sulfoxide (DMSO–SRL- Cat no.- 67,685) and read in an Elisa plate reader (iMark, Biorad, United States) at 540 nm. IC50 was calculated by using the software Graph Pad Prism −6. Images were captured under an inverted microscope (Olympus ek2) using camera (AmScope digital camera 10 MP Aptima CMOS) and the results were presented as Mean ± SEM.

### 2.11 Cell proliferation assay

Cell proliferation of the sintered and sterilized HAP and HNC reinforced HAP samples on the MG-63 cell line was determined by Alamar blue assay. The cells (5,000–8,000 cells/well) were cultured in 96 well plates for 24 h in DMEM (Dulbecco’s Modified Eagle Medium) medium supplemented with 10% FBS and 1% antibiotic solution at 37 °C with 5% CO_2_. Cells were treated at final concentrations of 1, 10, and 25 μM for 24 h 10 μL of the Alamar Blue reagent per 100 μL medium was added after 24 h and further incubated for 3–4 h. Finally, the plates was read at 540/590 nm using fluorescence ELISA Plate reader (Agilent BioTek Epoch 2).

## 3 Results and discussions

The biggest challenge in robocasting is ensuring consistent flow of the prepared ceramic paste to achieve successful print structures. In this work, the extrudability of the prepared ceramic paste was studied by varying the concentration of the HPMC binder. The printability of the ceramic pastes was achieved by adding different concentrations of the HPMC binder to Hydroxyapatite. Figure 1, (i) shows the extrusion of the ceramic pastes from the extrusion nozzle. The extrusion study showed that the ceramic paste with 1wt% of HPMC binder exhibited poor flow consistency due to its low viscosity. The extruded paste appeared thin and failed to hold a uniform cylindrical shape exhibiting severe necking or continuous dripping. Figure 1, (ii) (a) shows significant sagging of the extruded paste as it was unable to support its own weight. This ceramic paste had a dominant viscous nature. The polymer network of the 1wt% HMPC binder was too thin to hold the HAP particles in the suspension during the flow leading to a runny consistency. On the other hand, the paste with 1.5wt% HMPC binder showed an optimal balanced flow consistency. The binder provided adequate network entanglement and internal friction to guarantee a cohesive Bingham plastic behavior of the paste. The extrusion appeared straight, uniform, and maintained its diameter. The extrusion also exhibited a promising spanning ability by forming straight horizontal beams throughout the pillars as shown in Figure 1, (ii) (b). This indicates a viscoelastic rheological state of the ceramic paste. Further increasing the binder concentration to 2wt% produced a stubby paste that resisted flow. The high viscosity of the paste necessitated high pressure during extrusion. The paste was stiff to extrude leading to curling and snapping. The high yield stress of the paste was indicative of its dominant elastic nature. Figure 2a shows the relationship between viscosity and the applied shear rate for the ceramic pastes with different HPMC concentrations. All three ceramic pastes exhibited characteristic non-Newtonian shear thinning behaviour. The viscosity started at a high value of around 6,000 Pa s at low shear rates and dropped as the shear rate increased, reaching near zero at higher rates. The HPMC polymer creates a messy, complicated network that holds the ceramics particles in place rendering the paste highly viscous. When the paste is filled in a cartridge and a shear force is applied by pushing the paste through the nozzle, the polymer chains extricate and align themselves in the direction of the flow (del-Mazo-Barbara and Ginebra, 2021). This makes the paste slide without difficulty. Figure 2b gives a deeper view into the microstructural interactions between the HPMC polymer and the HAP ceramic particles under continuous flow.

**FIGURE 1:**
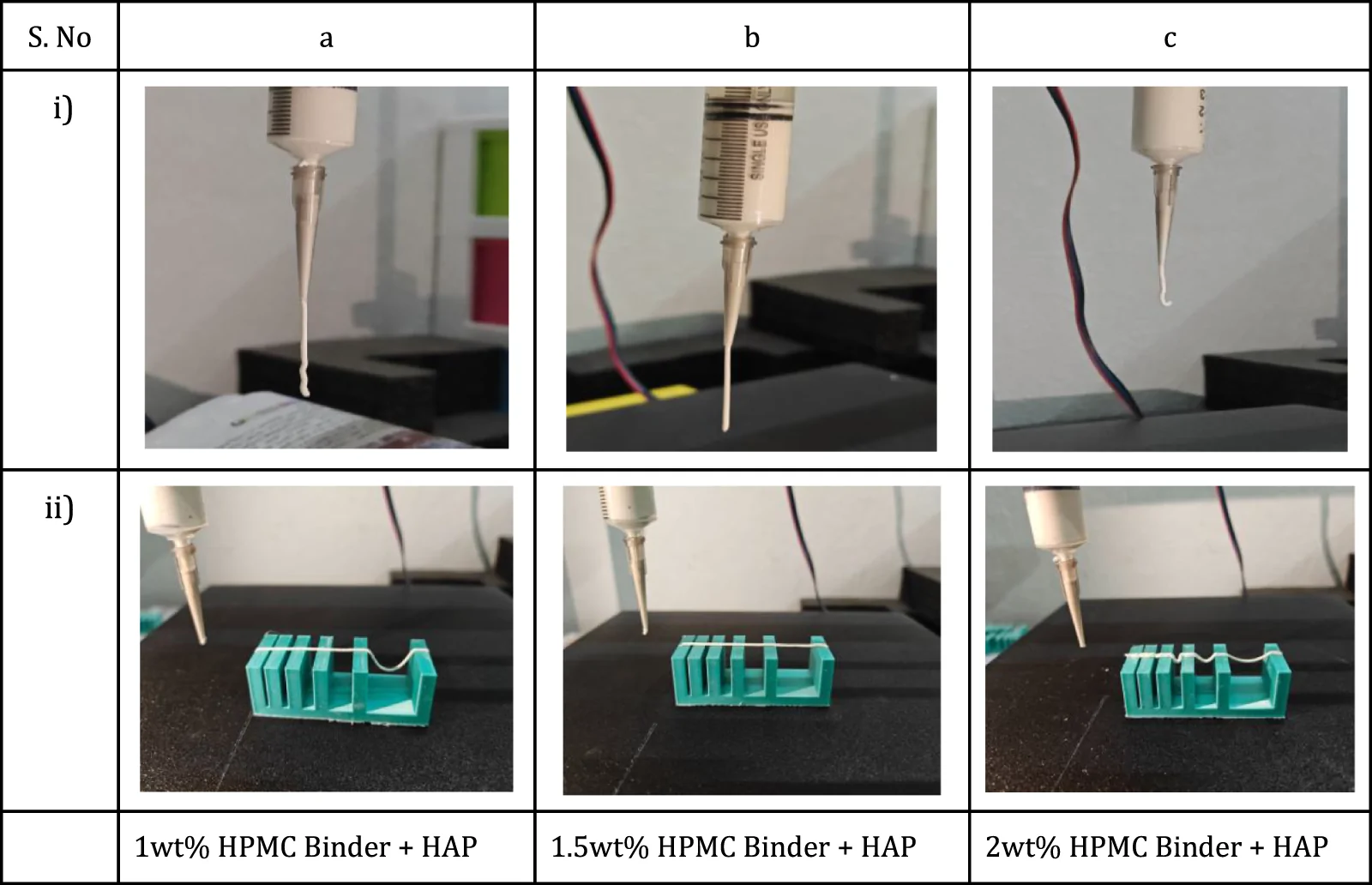
(i) The extrusion of **(a)** 1 wt.% HPMC Binder + HAP, **(b)** 1.5 wt% HPMC Binder + HAP and **(c)** 2 wt.% HPMC Binder + HAP slurry using a 16G nozzle and (ii) Strand deposition test performed for the prepared **(a)** 1 wt% HPMC Binder + HAP, **(b)** 1.5 wt% HPMC Binder + HAP and **(c)** 2 wt.% HPMC Binder + HAP.

**FIGURE 2:**
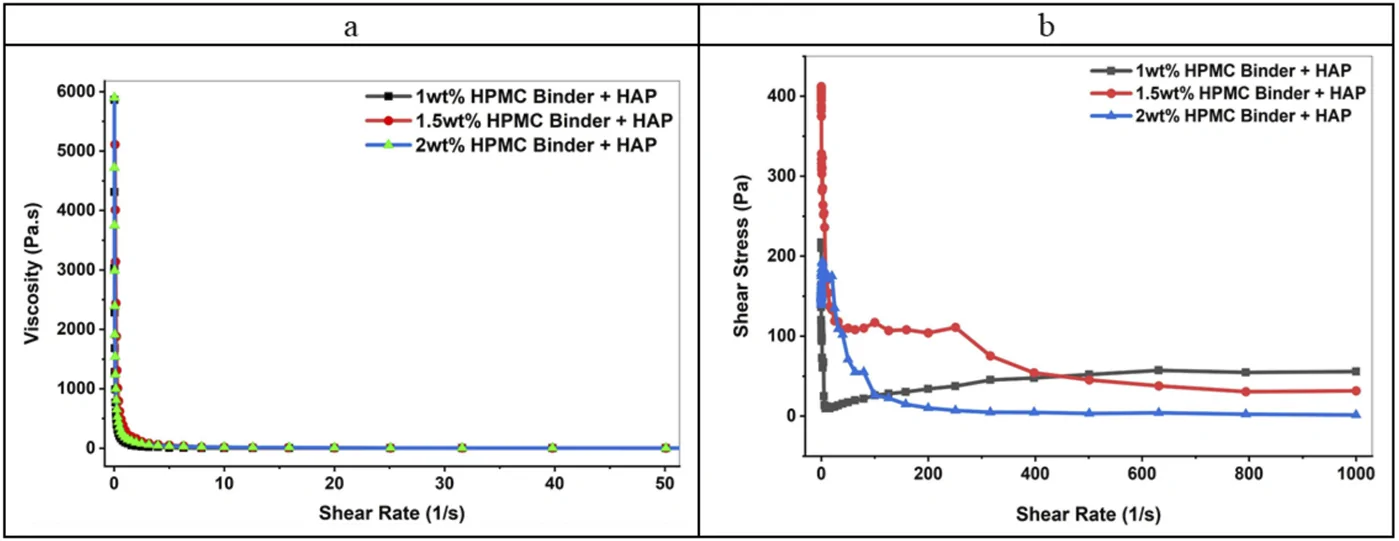
Rheological characterization of Ceramic pastes **(a)** Viscosity vs shear rate **(b)** Shear stress vs Shear rate.

The initial peaks (yield stress) in the figure indicate that some amount of force is required to collapse the structure of the ceramic pastes and initiate flow. The paste with 1.5wt% binder exhibited the highest initial yield stress spiking over 400 Pa. This demonstrates that the paste with 1.5wt% binder developed a sturdy and highly entangled network with the HAP ceramic particles at rest. The pastes with 1wt% and 2wt% binder concentrations exhibited lower yield stress with peaks around 200 Pa indicating an initial weaker static structure compared to that in the 1.5wt% paste. At high shear rates, the behavior of the ceramic paste significantly depended on the binder concentration. The shear stress in the 1wt% paste dropped after the initial yield and then gradually increased with increasing shear rate indicating mild shear thinning at higher speeds. Due to the low binder concentration, the particles collide and jam together resulting in hydrodynamic clustering and increased resistance to flow. While the 1.5wt% paste exhibited a high yield peak, its shear stress dropped and later stabilized at a lower value (Abu-Jdayil, 2011). This demonstrates that when a strong initial network is broken, it provides sufficient lubrication for consistent and smooth flow without any particle interruption. For the ceramic paste with 2wt% HPMC, a sudden drop in shear stress after yield was observed. The stress remained extremely low, almost close to zero, throughout higher shear rates. The high concentrations create slippers and are prone to suffer from structural collapse and shape retention problems after extrusion.

The microstructures and morphologies of the powder hydroxyapatite and halloysite nanoclay were examined using FESEM. The HAP had rod-like particles with irregular morphologies and dimensions as shown in Figure 3a. The rod-like hydroxyapatite is considered to be of significance and has been used in the field due to its structural similarity to the bones (Roopavath et al., 2019; Cascione et al., 2022). The HNC was observed as tubular structures with diameters ranging between 2–5 μm and 20–70 nm as shown in Figure 3b. Its tubular shape makes it unique and suitable for a wide range of uses. From the FESEM image the pores size of the sintered scaffold are evaluated using ImageJ. The strand of the scaffold contains multiple micropores and nanopores, these are the pores created between the HAP and HNC particles after the removal of binder during the heat treatment process (Ash, 2026). The micro pores were found to be between 0.1 µm and 6 µm and nanopores between 2 nm and 1 µm throughout every scaffold. This size is influenced by the particle size, sintering temperature and solid loading in the ceramic paste (Zhang et al., 2012). The observed surface properties result in an increase in the scaffolds surface area, which is beneficial for functionalities such as cell adhesion and exchange of waste materials and nutrients in biological environments. The mechanical and biological characteristics are optimized only if the reinforcement is uniformly distributed throughout the matrix. Homogeneous distribution of HNC reinforcement was observed in scaffolds with 1wt% and 2wt% HNC as shown in Figures 4b,c. In the HAP+3 wt% HNC scaffold, microcracks, clustering, and clumping of the reinforcing material were observed as in Figure 4d. These microcracks developed due to the stress produced by the agglomeration of excessively added reinforcement (Li et al., 2020). Hence, reinforcement concentrations were restricted between 1wt% and 3wt% in the present study. The EDS spectra of the sintered scaffolds were mapped at a magnification of ×100,000. The presence of Ca, P, and O were noticed in the pure hydroxyapatite scaffold as shown in Figure 4a, while peaks corresponding to Al and Si were seen in all the hydroxyapatite scaffolds reinforced with HNC. This shows the retention of HNC after high temperature sintering. Also, the presence of HNC in the sintered composite scaffolds confirmed through the Fourier transform IR spectroscopy (FTIR) as reported in our previous work (Ashwin et al., 2025). The existence of PO_4_^3-^ was evident at 511 cm^-1^ and 1,050 cm^-1^ peaks. The presence of and the CO_3_^2-^ peak was observed at 1,630 cm^-1^ and the water bond OH observed from 3,500 to 2,500 cm^-1^ (Huang et al., 2017). The stretching of Si-OH, Al-OH bonds and Si-O-Si bonds are observed at 480, 910 and 1,050 cm^-1^.

**FIGURE 3:**
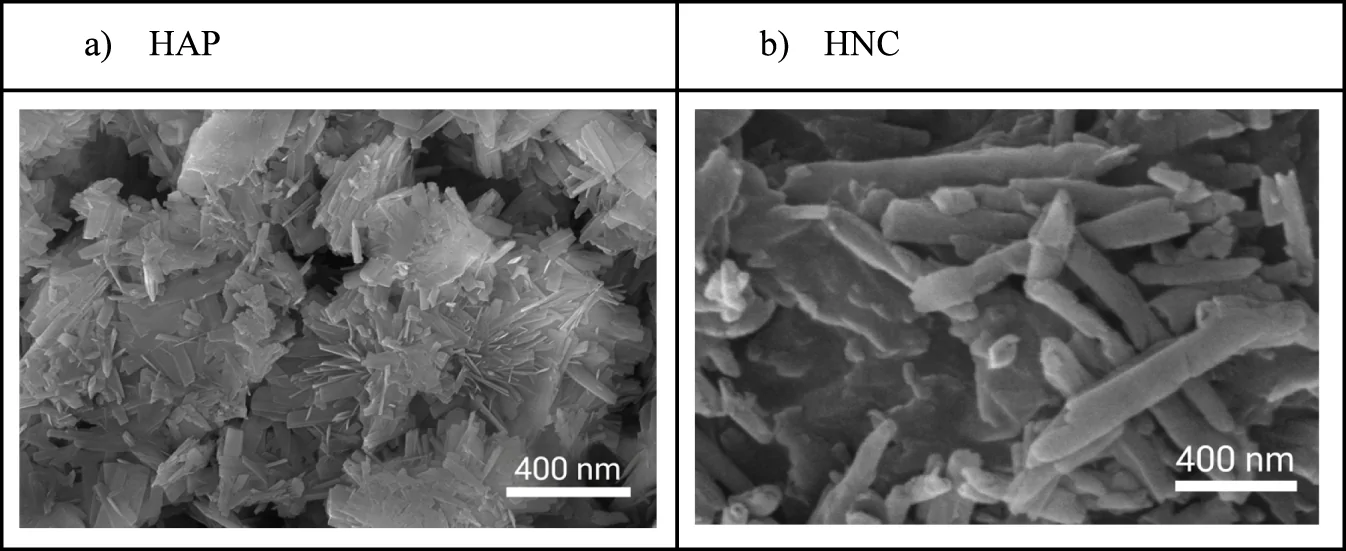
FESEM image of powder **(a)** HAP at 200,00x and **(b)** HNC at 2,000,00x.

**FIGURE 4:**
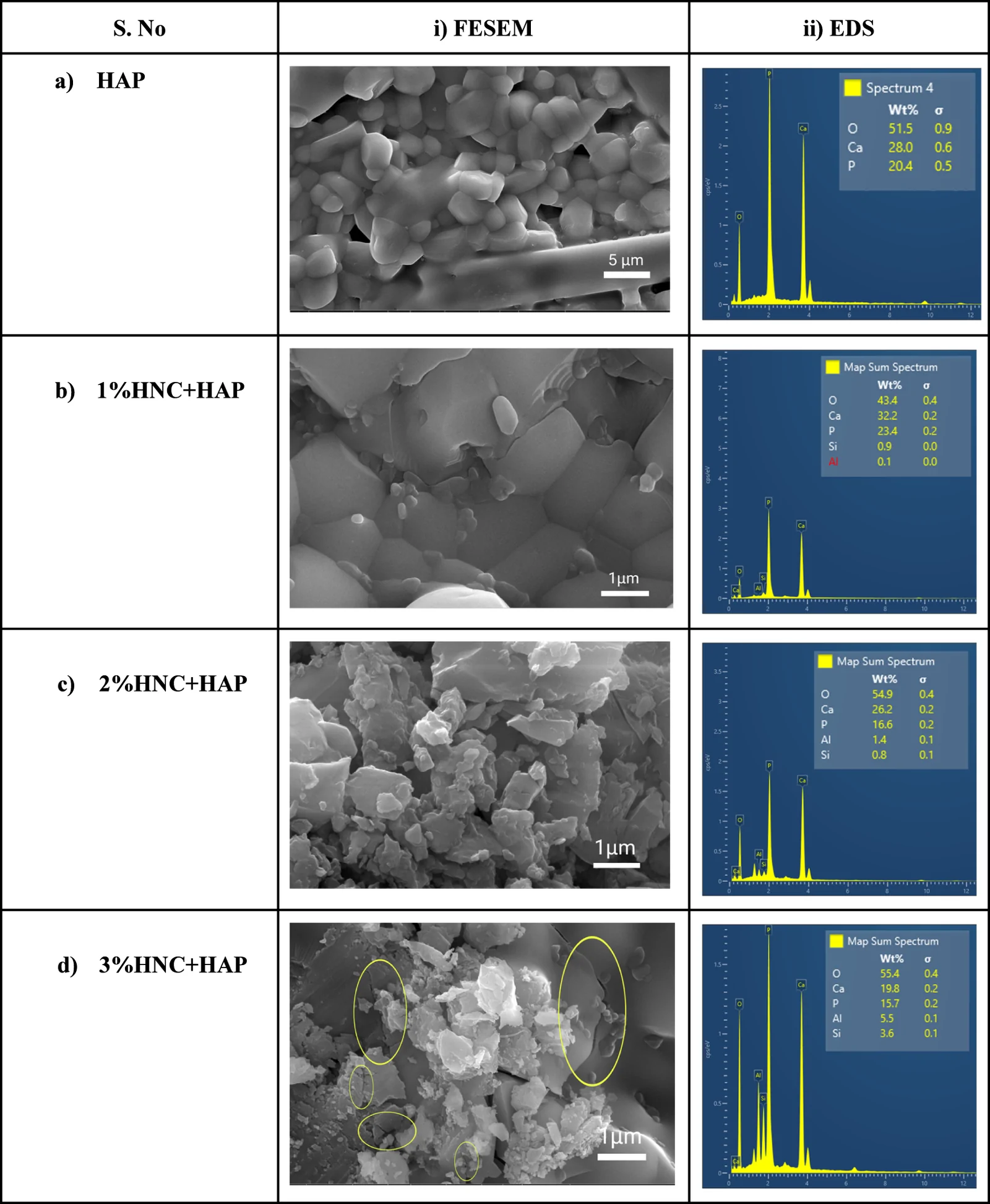
FESEM image and EDS spectra of sintered HAP and HNC reinforced Ceramic scaffolds **(a)** HAP scaffold at 100,00x, **(b)** 1%HNC + HAP, **(c)** 2%HNC + HAP, and **(d)** 3%HNC + HAP at 500,00×.

Figure 5a presents the Vickers microhardness (HV) values of the sintered hydroxyapatite scaffolds and halloysite nanoclay reinforced HAP nanocomposite scaffolds. The hydroxyapatite scaffold exhibited a baseline microhardness of 245.67 H V. The addition of 1wt% halloysite nanoclay produced a noticeable increment to 257.32 H V. With further increase in HNC concentration to 2 wt%, the microhardness improved to 271.67 H V. However, at 3 wt% of HNC, the microhardness improved only marginally to 274.67 H V. The initial increment in the Vickers hardness for scaffolds with HNC concentrations varying from 0 to 2wt% demonstrates that the HNC provides effective reinforcement to the HAP matrix. HNC possesses higher aspect ratio, a rigid one dimensional nanotubular geometry, and superior mechanical strength. When HNC disperses uniformly within the HAP matrix, the composite facilitates efficient load transfer from the weaker ceramic phase to the stronger reinforcing nanotubes (Topuz et al., 2021). A noticeable decline in the improvement rate of the microhardness at higher HNC concentration (>2wt%) indicates that the strengthening effect has reached a plateau. An ideal nanoclay dispersion is crucial for the scaffolds manufactured through the Robocasting technique and sintered at high temperature. During the sintering phase, the homogeneously mixed 1wt% and 2wt% HNC supports localized matrix densification. The HNC reinforcement bridges microcracks, deflects crack propagation, and absorbs fracture energy when the scaffold is subjected to mechanical loading. This directly reflects in their higher Vickers microhardness values. However, in the 3wt% HNC reinforced HAP scaffold, diagonal edge breakage was observed during testing, which can be attributed to the non-homogeneous mixing and agglomeration. Excess nanoclay in the matrix leads to agglomeration and formation of non-homogeneous clusters, which act as structural defects and stress concentrators. These agglomeration and formation of non-homogeneous clusters also affect the compressive strength of the scaffolds. From our previous work findings, HAP scaffolds exhibit 46.72MPa, the scaffolds with 2wt% shows 22% of improved compressive strength of 68.81 MPa. But the scaffold with highest 3wt% produced least 48 MPa among other composite scaffolds (Ashwin et al., 2025). The porosity percentage of sintered scaffold was evaluated by liquid displacement method using ethanol as a soaking agent (Mondal et al., 2020). The porosity was measured by weighing scaffolds in dry, wet and saturated condition. Then evaluated using Archimedes principle. The porosity percentage of sintered scaffolds are 39.18% (HAP), 38.82% (1HNC + HAP), 38.35% (2%HNC + HAP) and 38.21% (3%HNC + HAP). Figure 5b shows there is no major difference in the porosity percentage but the trend remains descending. This shows that increasing the HNC concentration will decrease the porosity of the scaffolds (Liu et al., 2015). The difference between the scaffold with 2wt% and 3wt% of HNC is very less compared with other combination. This may be due to the presence of microcracks in scaffold with 3wt% of HNC (Resorption and Weber, 2022).

**FIGURE 5:**
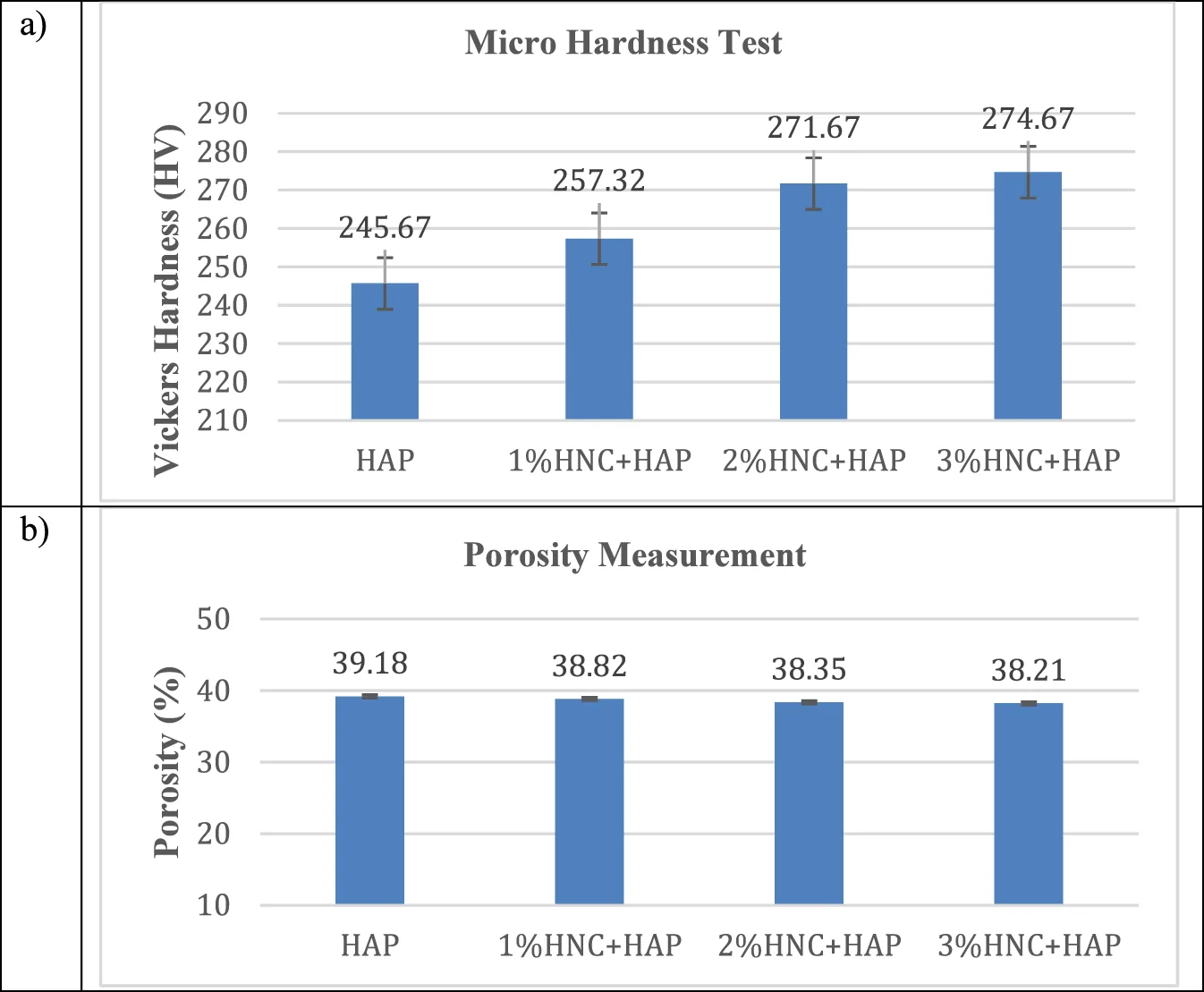
**(a)** Microhardness for sintered HAP and HNC reinforced HAP scaffolds, **(b)** Porosity measurement for sintered HAP and HNC reinforced HAP scaffolds.

From the swelling study the fluid uptake capacity of the HAP and HNC reinforced HAP scaffolds were measured at 12, 24 and 48 h time intervals. This ensures nutrient transport, cell migration and gas exchange ability of the scaffold (Mo et al., 2023). The scaffolds were immersed in PBS individually and placed in an incubator at 37 °C. Then the scaffolds taken out from the medium and wet weight are measured. For the first 12 h the uptake percentage increased quickly, 17.39% (HAP), 18.06% (1HNC + HAP), 18.24% (2%HNC + HAP) and 18.28% (3%HNC + HAP). Then the swelling slowed down between 12 and 24 h. After 24 h it reaches near saturation equilibrium as fluid fills the micropores and achieves a stable plateau between 48 h. Figure 6 shows no notable increment in the swelling ratio of 19.59 (1%HNC + HAP), 19.75 (2%HNC + HAP), 19.89 (3%HNC + HAP) scaffolds while comparing with 19.11 (HAP) scaffolds. But the minimal increments were absorbed due to the HNC’s layered structure that accommodates fluids. Due to the rigid crystal lattice of HAP, there is no volumetric expansion like polymeric hydrogels (Zhang et al., 2014). The reason for mass gain of the scaffolds is fluid uptake inside existing micro and macro pores. The scaffolds with lower swelling will exhibit slower degradation (Rianti et al., 2022). The degradation study from our previous work on HAP and HAP - HNC evident this by its slower degradation ability. During the initial phase day 1 to day 11 al l scaffolds showed mass gain from 0.56g (HAP) to (0.86g 3%HNC + HAP) due to the apatite layer formation on the scaffold surface. The gradual degradation occurs after day 11. Each scaffold exhibits slow and controlled degradation. The mass reduction is less than 5% after 3 weeks (Ashwin et al., 2025). The antimicrobial activity of the sintered HAP and HAP–HNC scaffolds was evaluated against *Staphylococcus aureus* (strain MTCC 452) by using the zone of inhibition (Kirby Bauer disk diffusion) method (Saha et al., 2010). For the antimicrobial activity study, the HAP and HAP–HNC composites were weighed accurately and highly concentrated dispersions, typically 100 mg/mL were prepared. Dimethyl sulfoxide was used as the solvent due to its excellent dissolving capacity for organic and inorganic compounds, as well as its lack of interference in antibacterial assays. Carefully weighed quantities of HAP and HAP–HNC samples were crushed and added to DMDO and sonicated to form a homogeneous suspension. As the HAP ceramic samples are not soluble in DMSO, the target is to create a uniform suspension unlike solution.

**FIGURE 6:**
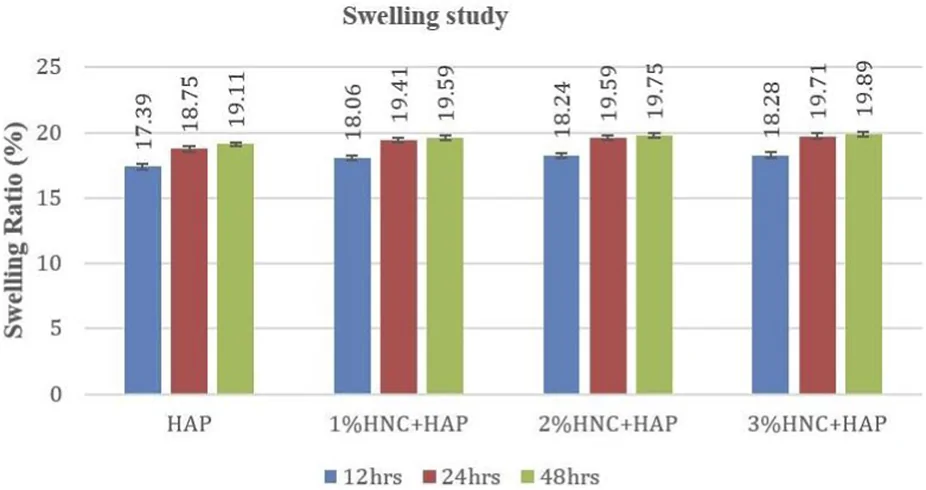
Swelling ratio (%) for sintered HAP and HNC reinforced HAP scaffolds.

To prepare a concentration of 100 mg/mL, 100 mg of the sample was dispersed in 1 mL of DMSO. Further dilutions were made in separate sterile tubes to prevent contamination. The 10 µL sample was placed on a 5 mm disc Whatman No1 filter paper using micropipette (Bediako et al., 2018). The discs were dried in an incubator at 37 °C for 30 min to completely remove DMSO, thus preventing solvent interference during the assay. Positive control was prepared by filling the disc with 10 µg of Ciprofloxacin (antibiotic) and the negative control was loaded with 10 µL of DMSO. The pure culture of *S. aureus* bacterial suspension (0.5 McFarland, 1.5 × 10^8 CFU/mL) was evenly distributed on the Mueller-Hinton agar plate surface. 10µL of samples at different concentrations (0–100 mg/mL) were placed onto the inoculated agar surface using 5 mm filter paper discs. Ciprofloxacin (10µg/disc) used as the positive control and dimethyl sulfoxide (DMSO) as the vehicle control. The plates were incubated for 24 h at 37 °C. Table 1 shows a detailed summary of the antibacterial activity of the sintered HAP and HNC reinforced HAP scaffolds, wherein the antibacterial efficacy was evaluated through the zone of inhibition measurement against *Staphylococcus aureus*. The zone of inhibition is an area of clear ring bordering as shown in Figure 7, the samples where bacteria cannot grow, indicating its antimicrobial effect. A larger zone corresponds to a stronger antimicrobial effect. Ciprofloxacin that was used as the positive control exhibited a maximum inhibition zone of diameter 25.33 mm diameter, demonstrating superior antimicrobial assay performance. Subsequently, HAP with a concentration of 500 μg exhibited a moderate zone of inhibition, 8.66 mm in diameter. This proves that the HAP itself has an intrinsic anti-bacterial activity. This is significant for applications such as in hard tissue where infection control is needed. The HAP reinforced with 1wt% HNC (125 μg) showed a minimal inhibition zone of 4 mm. On the other hand, HAP reinforced with 2wt% and 3wt% HNC exhibited slightly increased zones of 6 mm. Thus, both the pure HAP and HAP-HNC composite samples possess measurable antibacterial activity, as evident from the formation of inhibition zones despite the fact that their zones were smaller than that produced by an antibiotic like Ciprofloxacin due to their different fundamental mechanism. Ciprofloxacin is an antibiotic that directly influences bacterial DNA reproduction, whereas HAP and HAP-HNC scaffolds intimidate bacteria through surface contact mediated mechanisms, electrostatic interactions and nano topographical stresses (Shariati et al., 2022). This is strong enough to disrupt initial bacterial colonization. In hard tissue engineering the localized and contact active antimicrobial response is enough for prophylactic prevention of biofilm, with uncompromised osteoblast viability and bone regeneration (Pant et al., 2019).

**TABLE 1:** *In vitro* antimicrobial assay showing the zone of Inhibition (mm) of ciprofloxacin, HAP and HNC reinforced HAP, scaffolds against *Staphylococcus aureus*.

S. No	Samples	Effective Qty (µg)	Zone of inhibition (mm)^a^
1	Ciprofloxacin	10	25.33 ± 0.33
2	HAP	500	8.66 ± 0.33
3	1%HNC + HAP	125	4.00 ± 2.00
4	2%HNC + HAP	250	6.00 ± 0.00^b^
5	3%HNC + HAP	125	6.00 ± 0.00^b^

a Values are presented as mean ± Standard Error of the Mean (SEM) (n = 3).

b An SEM, value of 0.00 indicates identical measurements across all triplicate trials.

**FIGURE 7:**
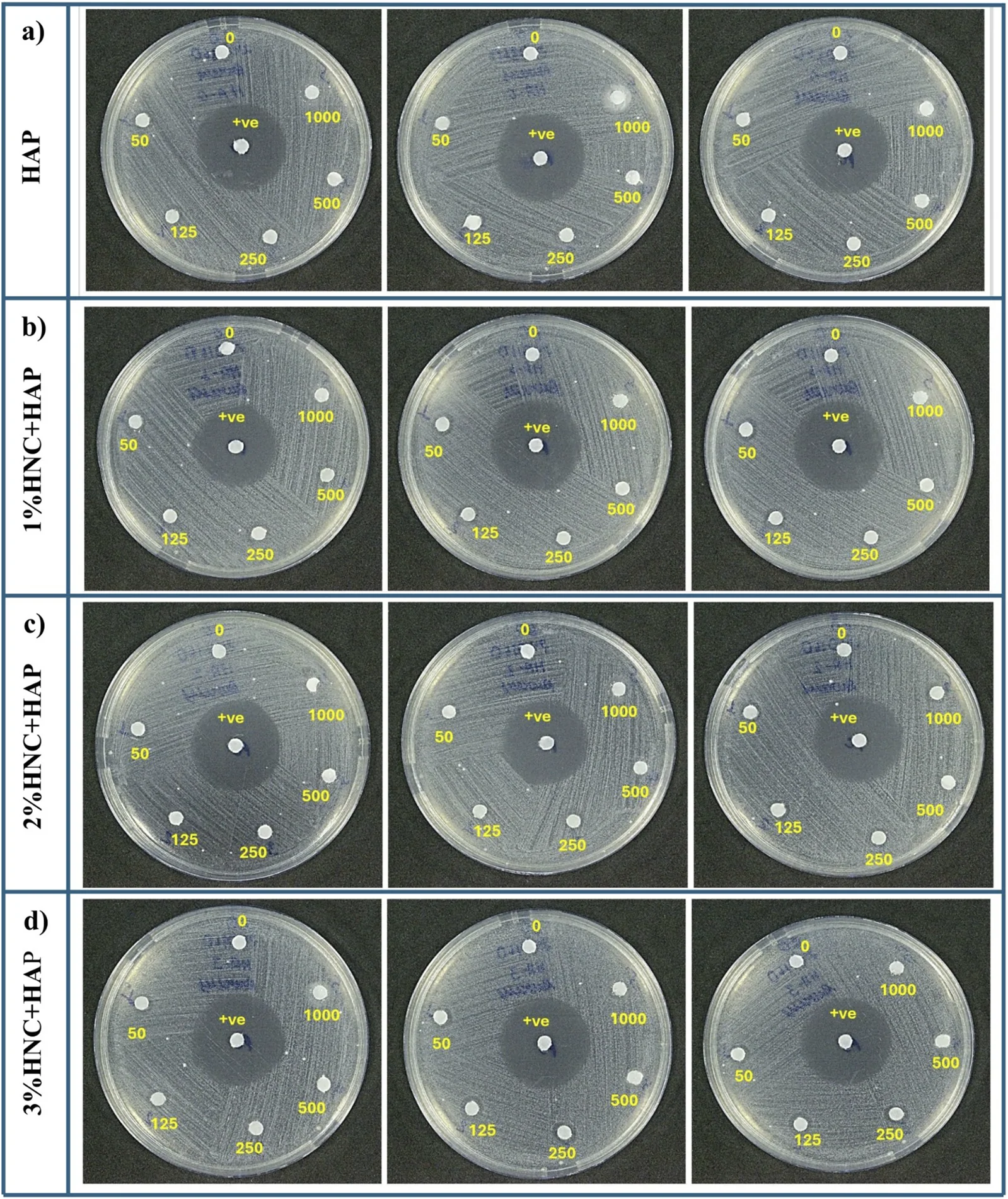
Antimicrobial assay **(a)** HAP, **(b)** 1%HNC + HAP, **(c)** 2%HNC + HAP, **(d)** 3%HNC + HAP.

The results evidently show that the HAP and HAP–HNC composites possess some inherent antibacterial properties. This inherent antibacterial property is important because the HAP and HAP-HNC can produce sustained antimicrobial effects without the risk of developing bacterial resistance. The antimicrobial (zone of inhibition) study demonstrates that the HAP and HAP-HNC composites are suitable for tissue engineering applications where moderate antibacterial properties are required. The *in vitro* cell viability assay study was conducted in HAP and HNC reinforced HAP scaffolds to evaluate their cytocompatibility. Figure 8 clearly indicates that HAP and HNC reinforced HAP scaffolds are non-toxic to the MG-63 cells (Hassanain et al., 2024). The addition of HNC to HAP was observed to enhance the cell viability due to the presence of Si ions (Porter et al., 2003). The x-axis in the graph represents the concentration of our material in µg/mL, while the y-axis denotes percentage of cell viability. For the pure HAP scaffold, the cell viability gradually decreased from 100% to about 71%–80%, with increasing concentration, indicating a mild, concentration-dependent reduction in metabolic activity with a relatively good viability (>70%).​ Even though higher doses slightly reduce cell proliferation/metabolic activity, HAP is generally cytocompatible with MG-63 cells. In HAP with 1%HNC, viability remained higher than that of pure HA across all concentrations (about 87%–95% at low–mid doses and ∼74% at the highest concentration), showing that HNC incorporation mitigates the drop-in cell activity at comparable doses, particularly in the low to mid concentration range, where the curve indicates better cytocompatibility than pure HAP.

**FIGURE 8:**
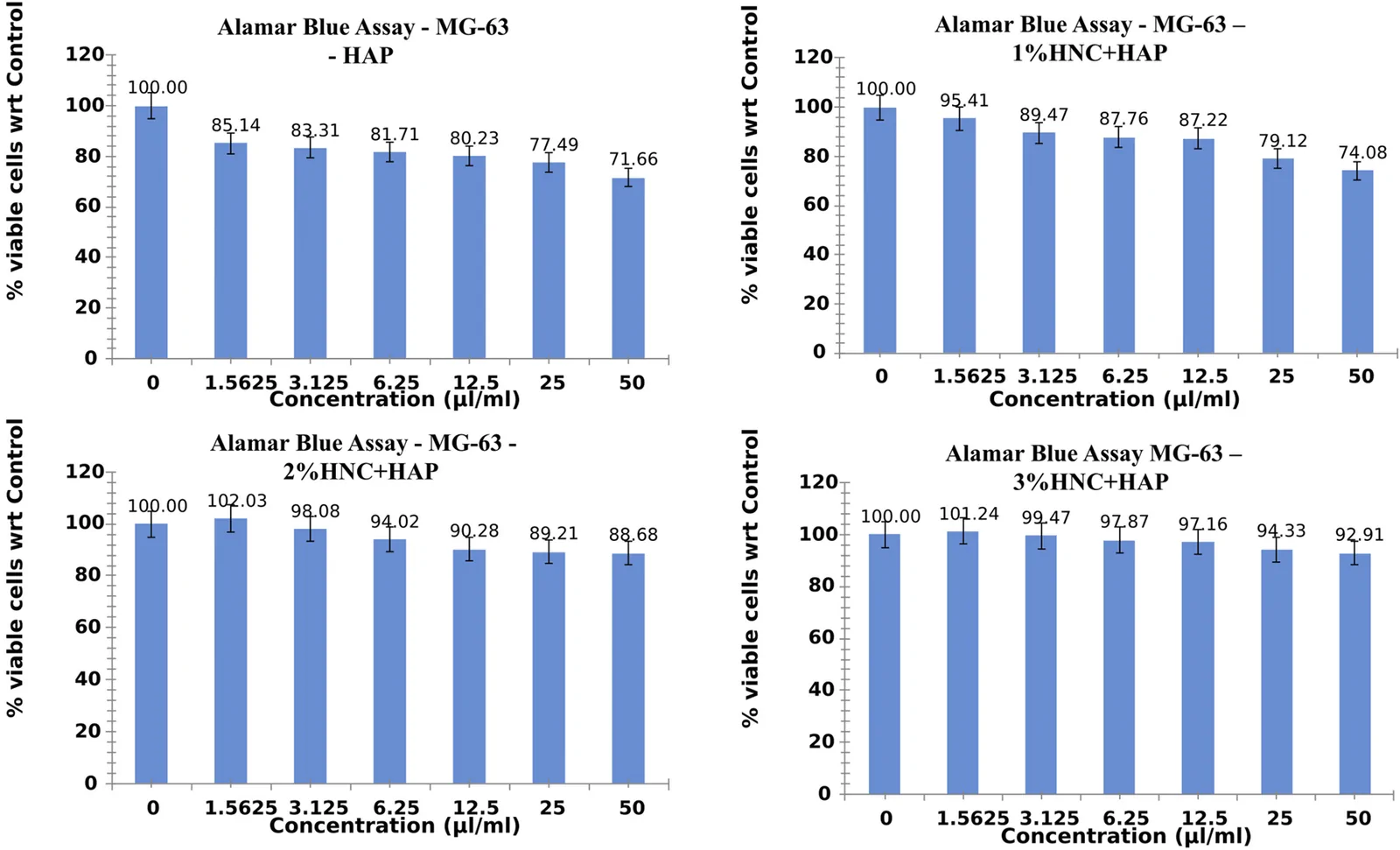
*In vitro* cell viability assay of **(a)** HAP, **(b)** 1%HNC + HAP, **(c)** 2%HNC + HAP, **(d)** 3%HNC + HAP, Scaffolds on MG-63 osteoblast cell line.

With the incorporation of 2wt% HNC, the viability was observed to be around or slightly above 100% at low concentrations and remained in the high range (80%–90%) even at the highest dose, indicating that this composition not only avoids cytotoxicity but may slightly stimulate MG-63 metabolic activity at low concentrations. At 3wt% HNC, the viability of the composite stayed very close to or slightly above 100% across most of the concentrations, dropping only modestly to about 93%–94% at the highest doses, demonstrating excellent cytocompatibility and possible pro-proliferative behaviour.​ All formulations were non-cytotoxic, with the MG-63 cell viability remaining mostly above 70%–80% even at the highest concentrations, confirming good *in vitro* biocompatibility. The Alamar Blue assay showed high viability for all HAP and HAP–HNC composites and a visibly increasing trend with increasing HNC concentration (HAP <1%HNC + HAP <2%HNC + HAP ≈3%HNC + HAP) indicating that HNC incorporation improves the biological response of the cells. Higher HNC loadings (2wt% and 3wt% HNC) provided the best cell tolerance and showed potential to better support cell proliferation. The cell proliferation assay was conducted using MG-63 osteoblast cells. These cells were treated with HAP and HNC reinforced HAP scaffolds at concentrations of 1, 10, and 25 μM. The samples were incubated for 24 h. The Alamar Blue assay revealed that the cells maintained high viability across all concentrations compared to untreated controls, indicating good cytocompatibility of the tested nanocomposites. This assay was conducted on 4 scaffolds-pure HAP scaffold and 1wt%, 2wt%, and 3wt% HNC reinforced HAP scaffolds. Figure 9 demonstrates that for the pure HAP scaffold, the cell viability decreased from 91.63% at 75 μg/mL down to 79.69% at 600 μg/mL. The viability of 91.63% at the lowest concentration indicates good compatibility. The viability of the scaffold with 1wt% of HNC was slightly higher than that of the pure HAP scaffold, ranging from 92.42% (75 μg/mL) to 82.43% (600 μg/mL). The 2wt% HNC reinforced HAP scaffold showed further improvement in viability with values ranging from 97.82% at 75 μg/mL to 84.74% at 600 μg/mL. The HAP scaffold with 3wt% of HNC reinforcement exhibited the highest cell viability across all concentrations peaking at 100.34% at 75 μg/mL. The enhancement of biological performance in 2wt% and 3wt% HAP-HNC scaffolds is observed when compared with other combinations of scaffolds due to the combination of biochemical signaling and microstructural optimization. The controlled release of biologically active SI ions from HNC promotes the proliferate pathways and cellular metabolism in cells (Makarova et al., 2023). Also, the reinforcement of HNC enhances a nano surface roughness (Lampropoulou and Papoulis, 2021) and improves overall scaffold hydrophilicity (Falcón et al., 2015), this enhances protein absorption and improves cell attachment (Yadav and Verma, 2023). From this discussion the Vickers hardness, swelling, porosity and *In vitro* studies evident that the scaffold with 2wt% of HNC reinforcement is suitable for repairing cancellous bone tissue (González-Alva et al., 2025).

**FIGURE 9:**
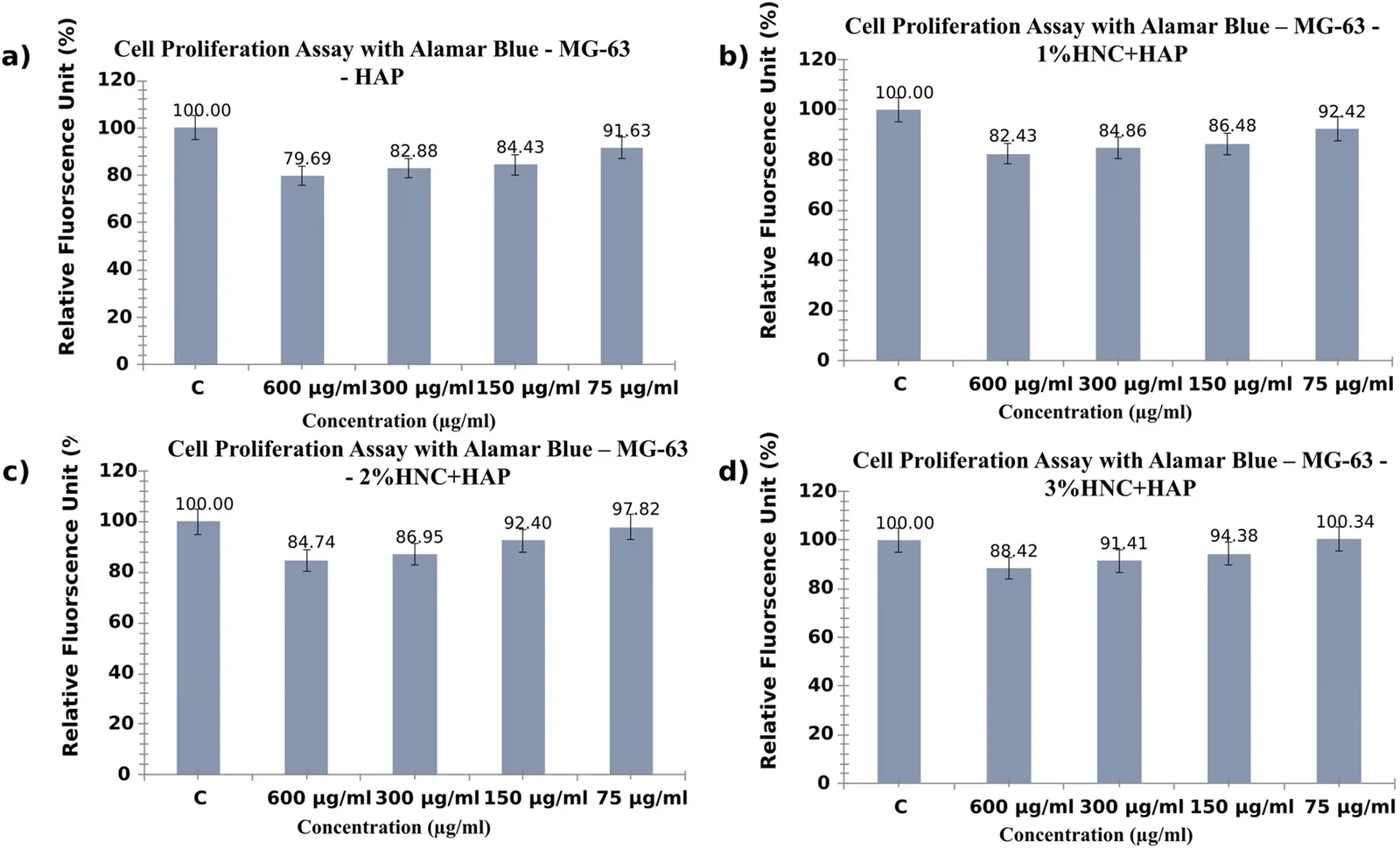
*In vitro* cell Proliferation assay of **(a)** HAP, **(b)** 1%HNC + HAP, **(c)** 2%HNC + HAP, **(d)** 3%HNC + HAP, Scaffolds on MG-63 osteoblast cell line.

## 4 Conclusion

This work effectively illustrates the fabrication of a new composite scaffold for bone tissue engineering, presenting an optimal balance between robocasting printability and the structural and biological performance. The research identified the right composition to fabricate mechanically stable, porous, and cytocompatible scaffolds by incorporating rod-like Hydroxyapatite and tube-like Halloysite Nanoclay within the Hydroxypropyl methylcellulose polymer binder framework. The printability and structural fidelity of the robocast scaffolds were controlled by the viscoelastic behavior dictated by the HPMC binder concentration. The paste with 1.5 wt% HPMC exhibited an ideal Bingham plastic behavior with a strong initial yield stress of 400 Pa, that provides the required internal friction and polymer entanglement for continuous and homogeneous extrusion with good post deposition shape retention without sagging or necking. On the other hand, the quality of the prints was found to be strongly affected at extreme concentrations of the binder. Low binder concentration of 1 wt% led to a more fluid-like and low viscous paste that tended to drip and collapse under its own weight, whereas high binder concentration of 2 wt% led to a highly elastic paste that required high extrusion pressures and was prone to structural snapping and geometrical defects. The geometrical synergy of the rod-like HAP and high-aspect-ratio HNC nanotubes greatly enhanced the physical attributes of the composite. The uniform dispersion of HNC at low to moderate loadings (1wt% and 2wt%) leads to efficient load transfer through crack-bridging, deflection, and local densification during sintering, causing a significant increase in Vickers hardness from 245.7 H V for pure HAP to a peak optimal threshold of 271.7 H V at 2 wt% HNC. However, a further rise in the HNC concentration to 3 wt% led to the paste hitting a critical dispersion limit with reduced mechanical returns (274.7 H V). At such high concentration, particle clustering and agglomeration occur creating significant stress concentrators that cause microcracks during binder burnout and sintering. A distinct microstructural limit for structural integrity was established at 2 wt%. The distinctive microstructural and compositional properties of the sintered scaffolds manifest themselves in terms of biological tolerance and preventive infection management. The sintering process created a favorable network of microporosity (∼6 μm) and surface roughness that directly aids cellular integration. The densification during high temperature sintering restricts the scaffold porosity less than 39%, this gives the optimal balance between mechanical strength and bioactivity. *In vitro* studies on MG-63 cell line show the absolute non-cytotoxicity of these scaffolds, with the presence of HNC actively favoring cell viability and metabolic activity, leading to the highest cellular tolerance in the 2wt% and 3wt% nanocomposites. The lower swelling ability is observed from swelling study this make the scaffolds slower degradation. At the same time, the scaffolds show a slight inherent antibacterial activity with 4–6 mm zones of inhibition. While this baseline antibacterial activity is less than commercial antibiotics, it still provides a beneficial layer of protection against localized implant infections, free from the strong selective pressure that often fuels antibiotic resistance. Among the different scaffolds examined in this study, the 2 wt% HNC + HAP formulation has emerged as the best composition for bone tissue engineering and orthopedic applications. Therefore, from the observed mechanical and biological properties, the scaffold with 2wt% HNC is more suitable for repairing the cancellous bone.

## Data Availability

The original contributions presented in the study are included in the article/Supplementary Material, further inquiries can be directed to the corresponding author.
