# Supplementary material for: Advanced fabrication and characterization of robocasted halloysite-hydroxyapatite nanocomposite scaffolds for hard tissue regeneration
Source: Front Bioeng Biotechnol. 2026 Oct 21;14:1909411. doi: 10.3389/fbioe.2026.1909411 (PMC13731216; doi:10.3389/fbioe.2026.1909411)
Supplement: Supplementary file 1 [file DataSheet1.pdf]

The swelling ratio of the HAP scaffold and HNC reinforced HAP scaffolds

S.NO	Sample	Time Interval (in Hours)					
		12hrs		24hrs		48hrs	
		%	SD	%	SD	%	SD
1	HAP	17.39	0.31	18.75	0.33	19.11	0.3
2	1%HNC+HAP	18.06	0.13	19.41	0.15	19.59	0.12
3	2%HNC+HAP	18.24	0.08	19.59	0.07	19.75	0.04
4	3%HNC+HAP	18.28	0.03	19.71	0.08	19.89	0.06
