# Supplementary material for: Advanced fabrication and characterization of robocasted halloysite-hydroxyapatite nanocomposite scaffolds for hard tissue regeneration
Source: Front Bioeng Biotechnol. 2026 Oct 21;14:1909411. doi: 10.3389/fbioe.2026.1909411 (PMC13731216; doi:10.3389/fbioe.2026.1909411)
Supplement: Supplementary file 2 [file DataSheet2.pdf]

24D16D

Antibacterial Zone Inhibition Test

Disc Diffusion Method

Test organism- *S. aureus*

Sample Code – HA 0

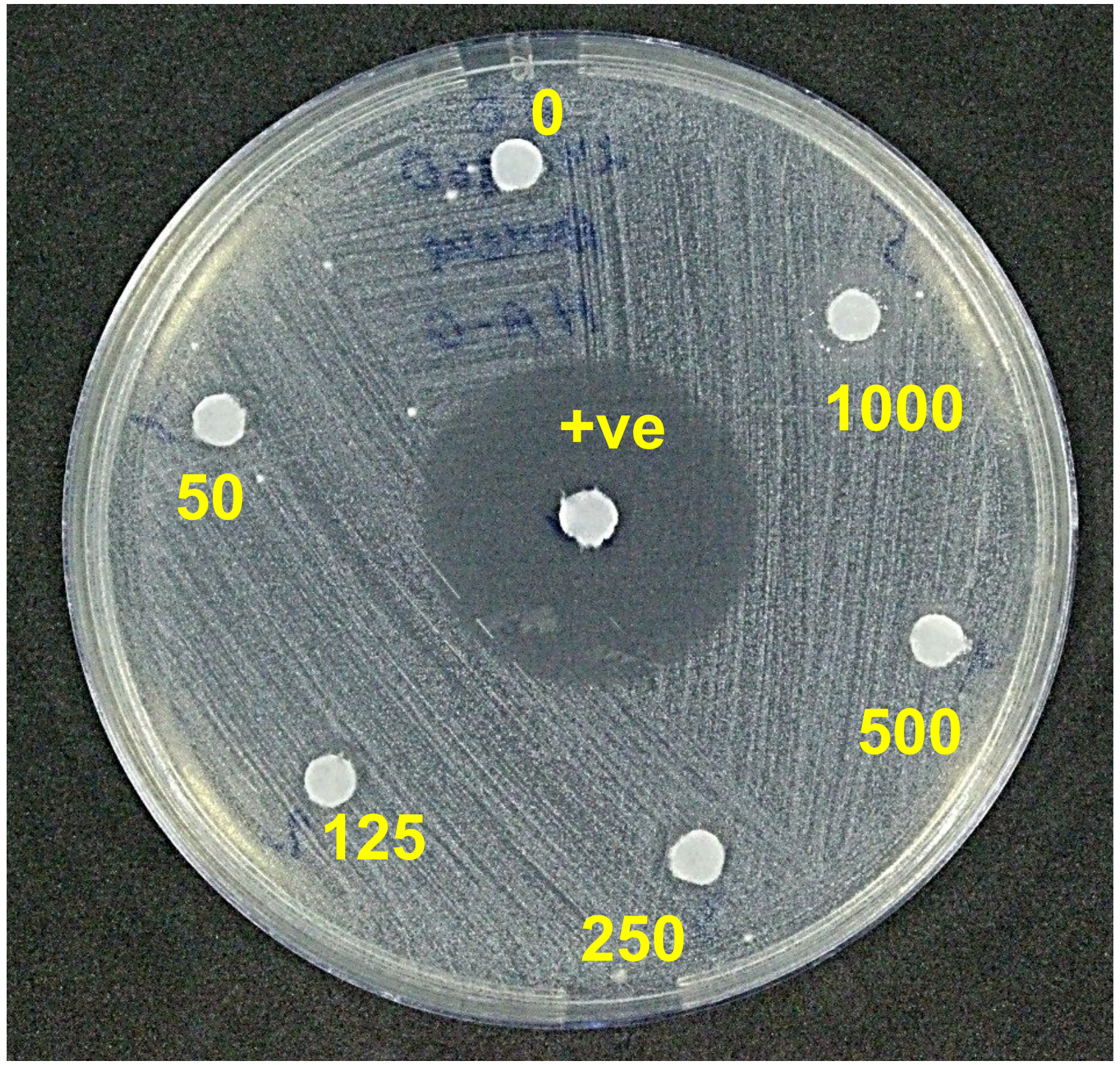

A

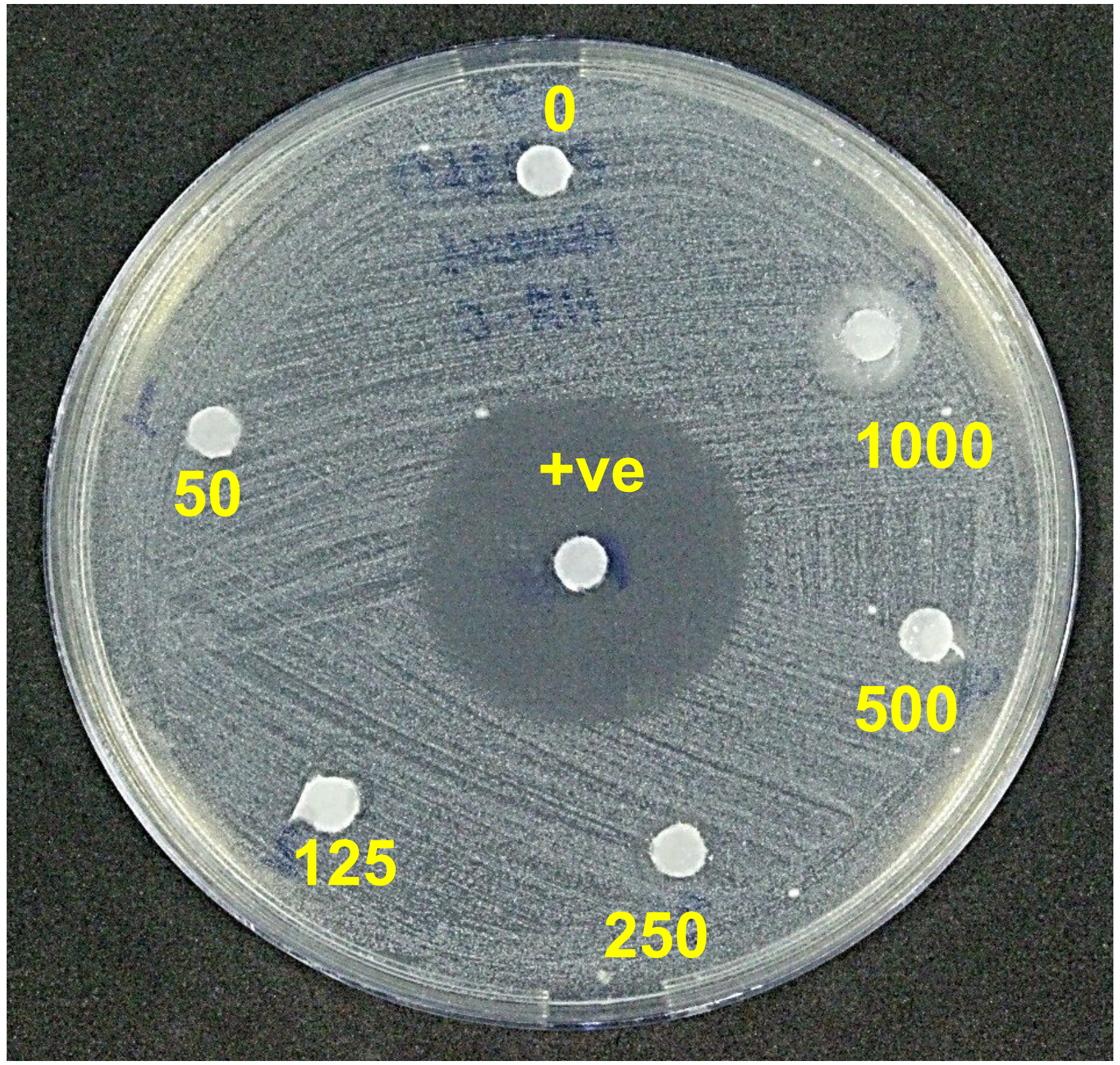

B

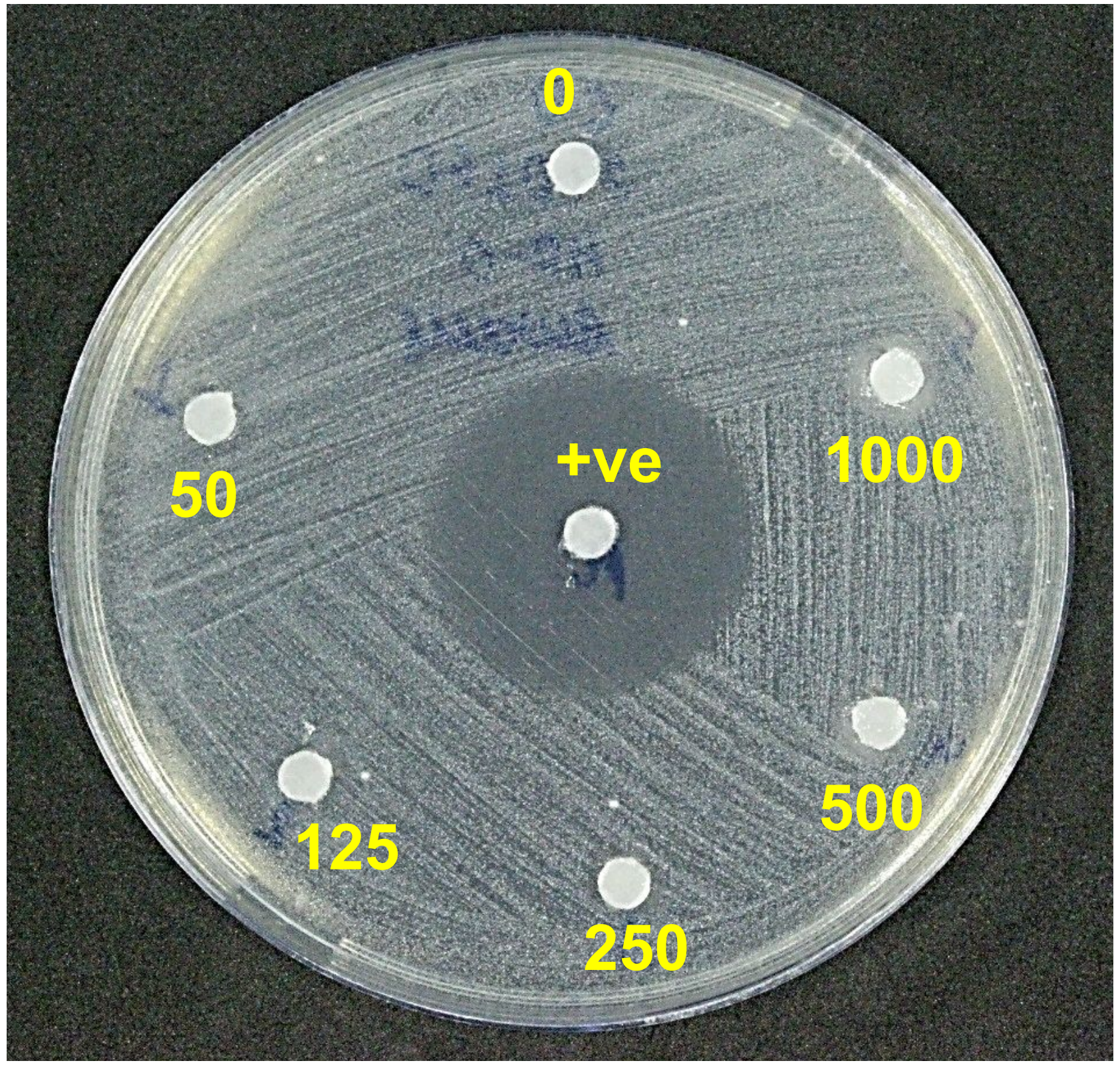

C

Amount present per disc in µg
Dispensed Volume - 10 µl
Positive Control (Ciprofloxacin) - 10 µg

Sample Code – HA 1

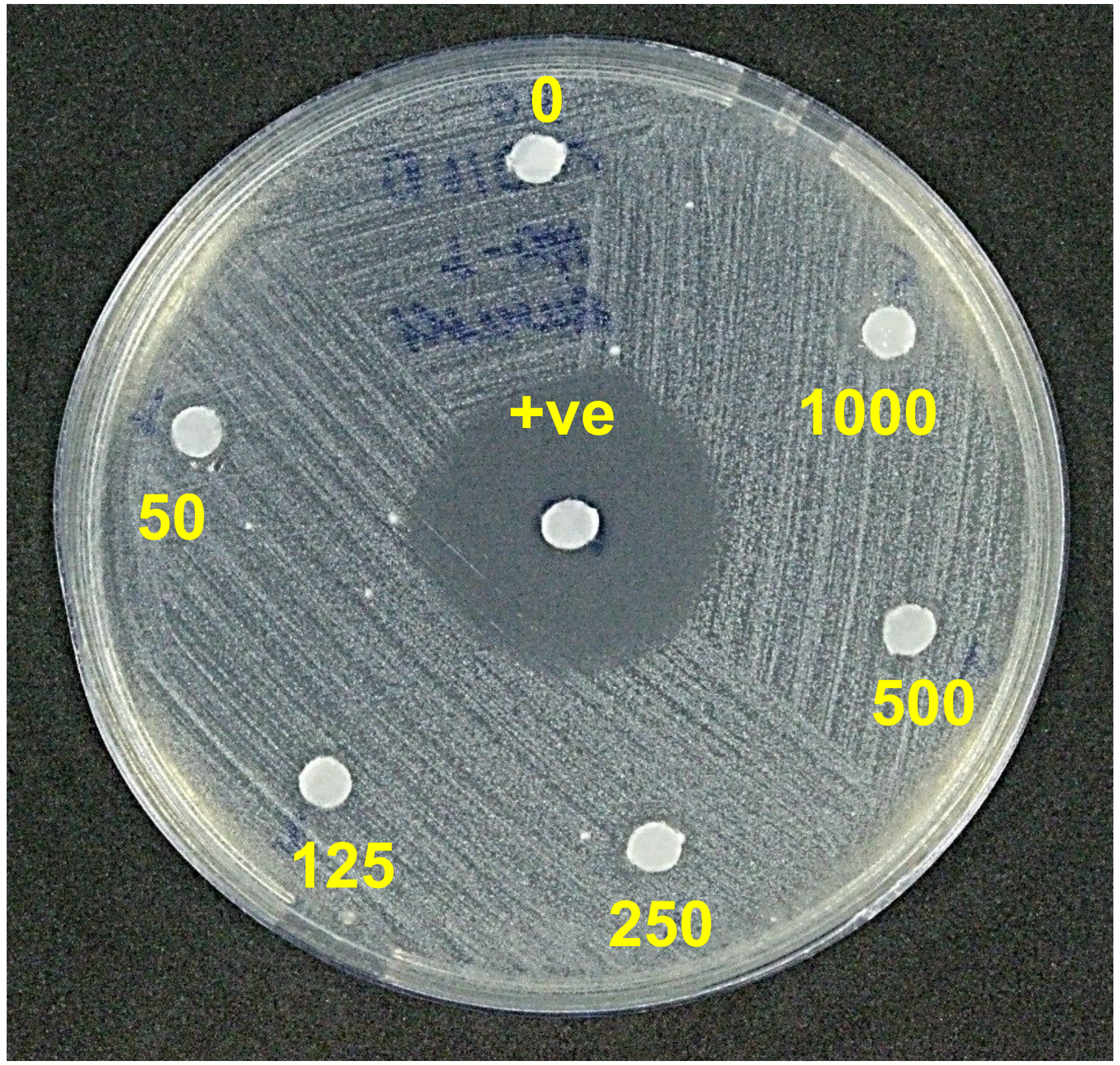

A

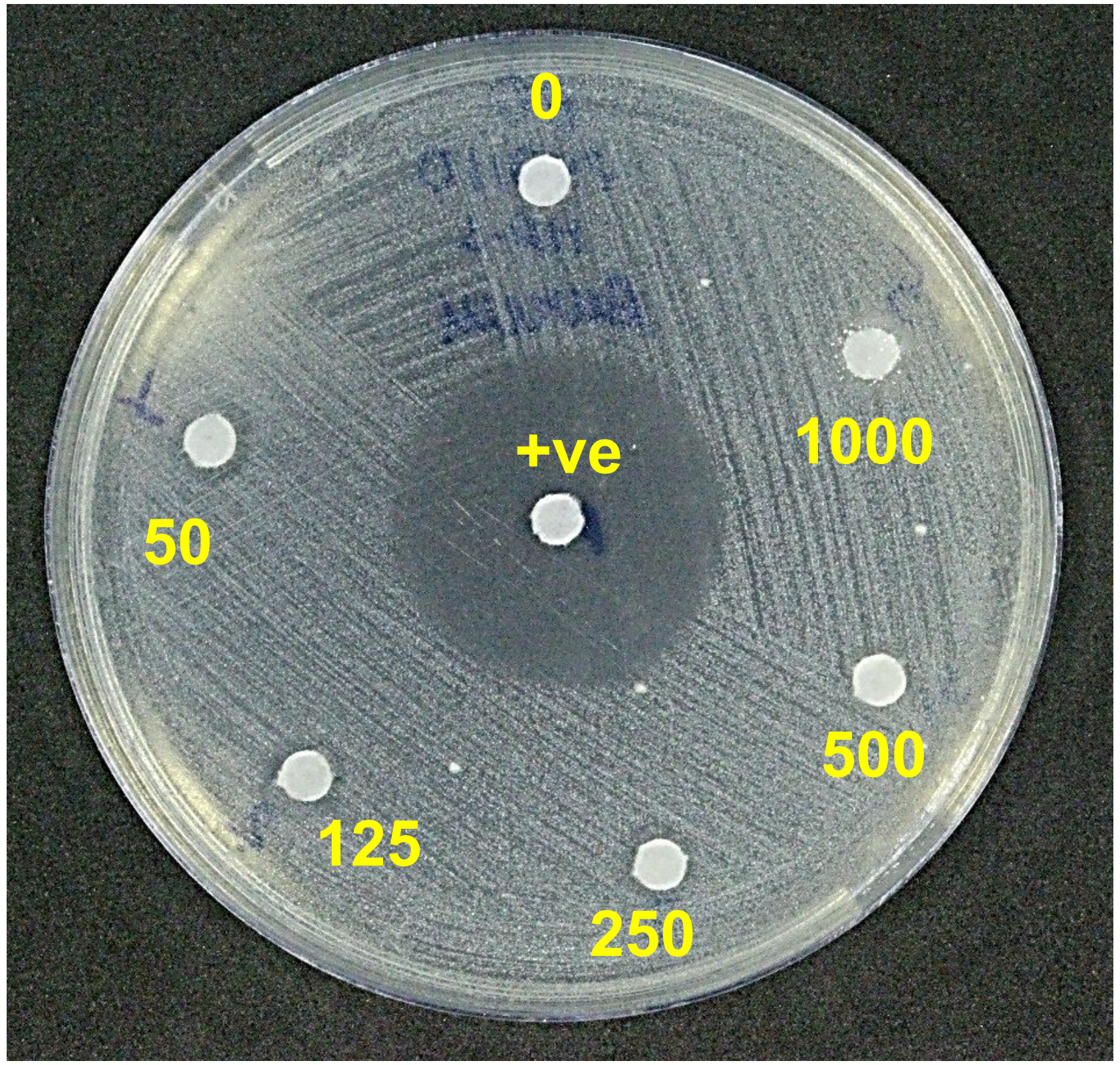

B

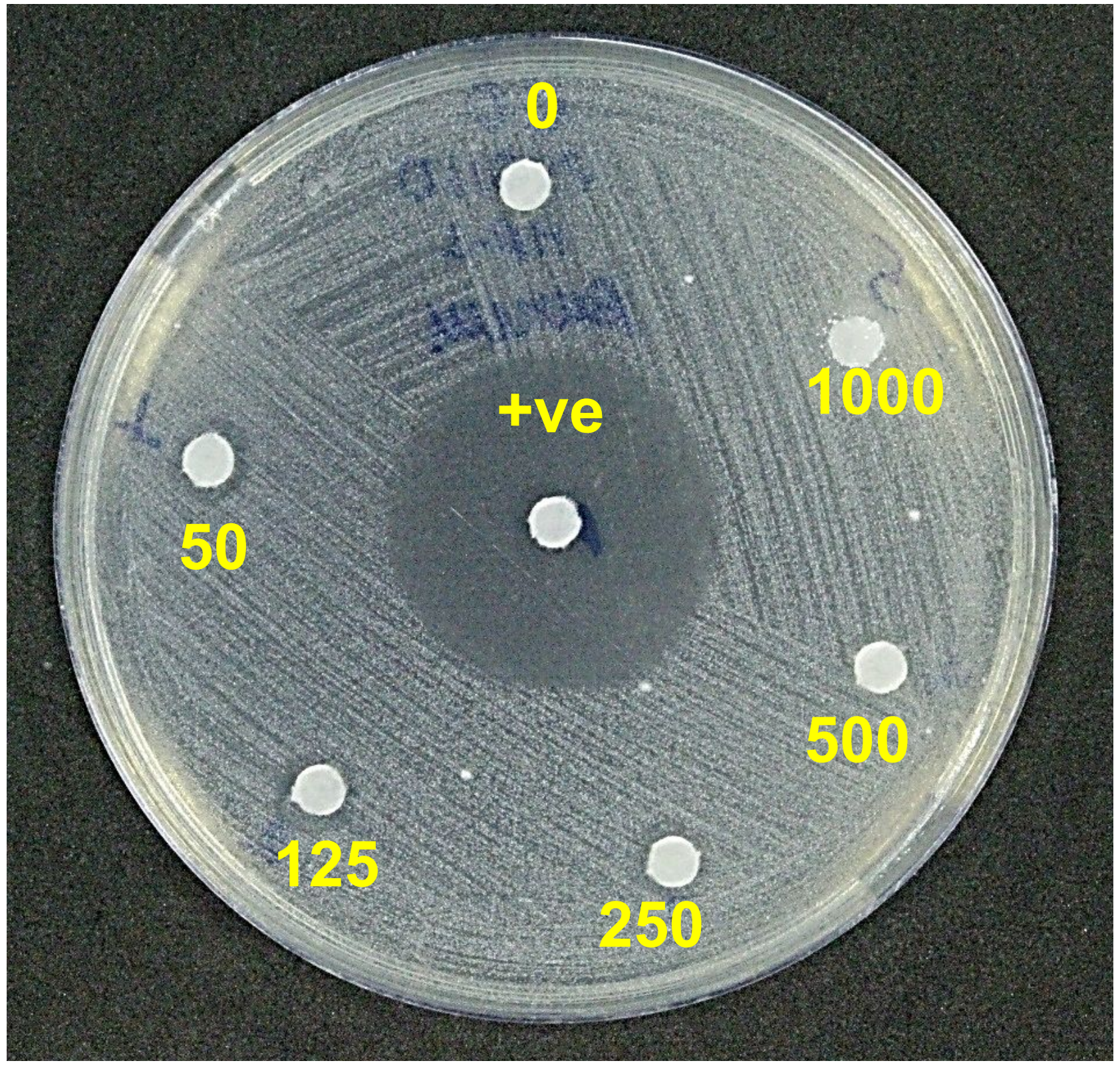

C

Amount present per disc in µg
Dispensed Volume - 10 µl
Positive Control (Ciprofloxacin) - 10 µg

Sample Code – HA 2

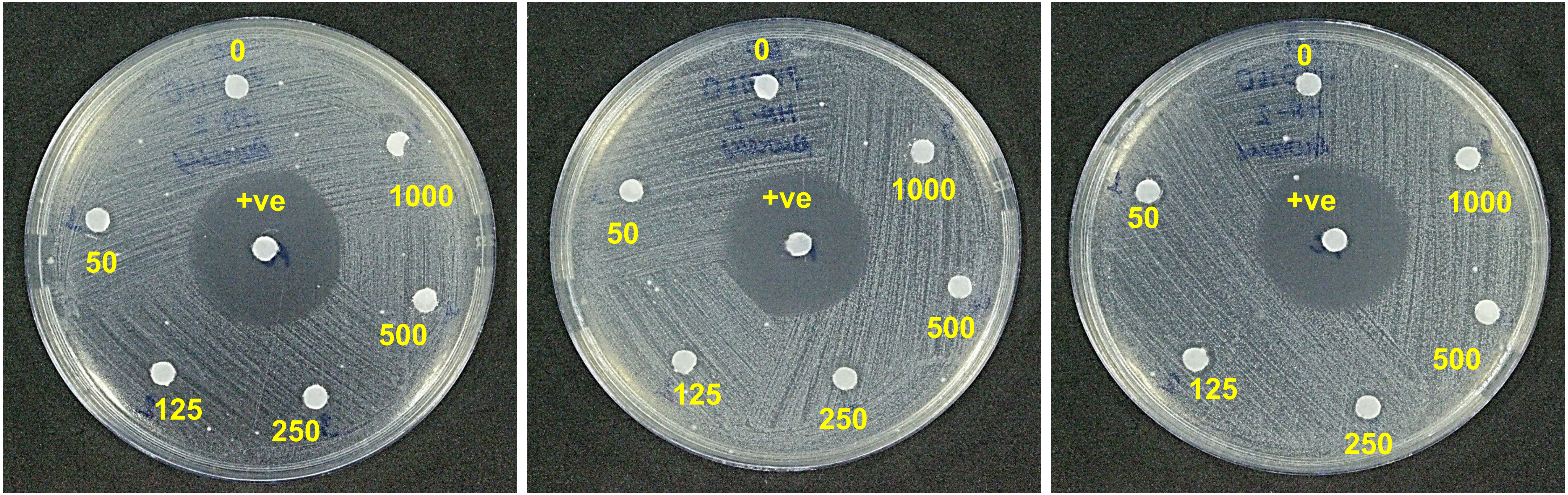

A **B** **C**

Amount present per disc in µg
Dispensed Volume - 10 µl
Positive Control (Ciprofloxacin) - 10 µg

Sample Code – HA 3

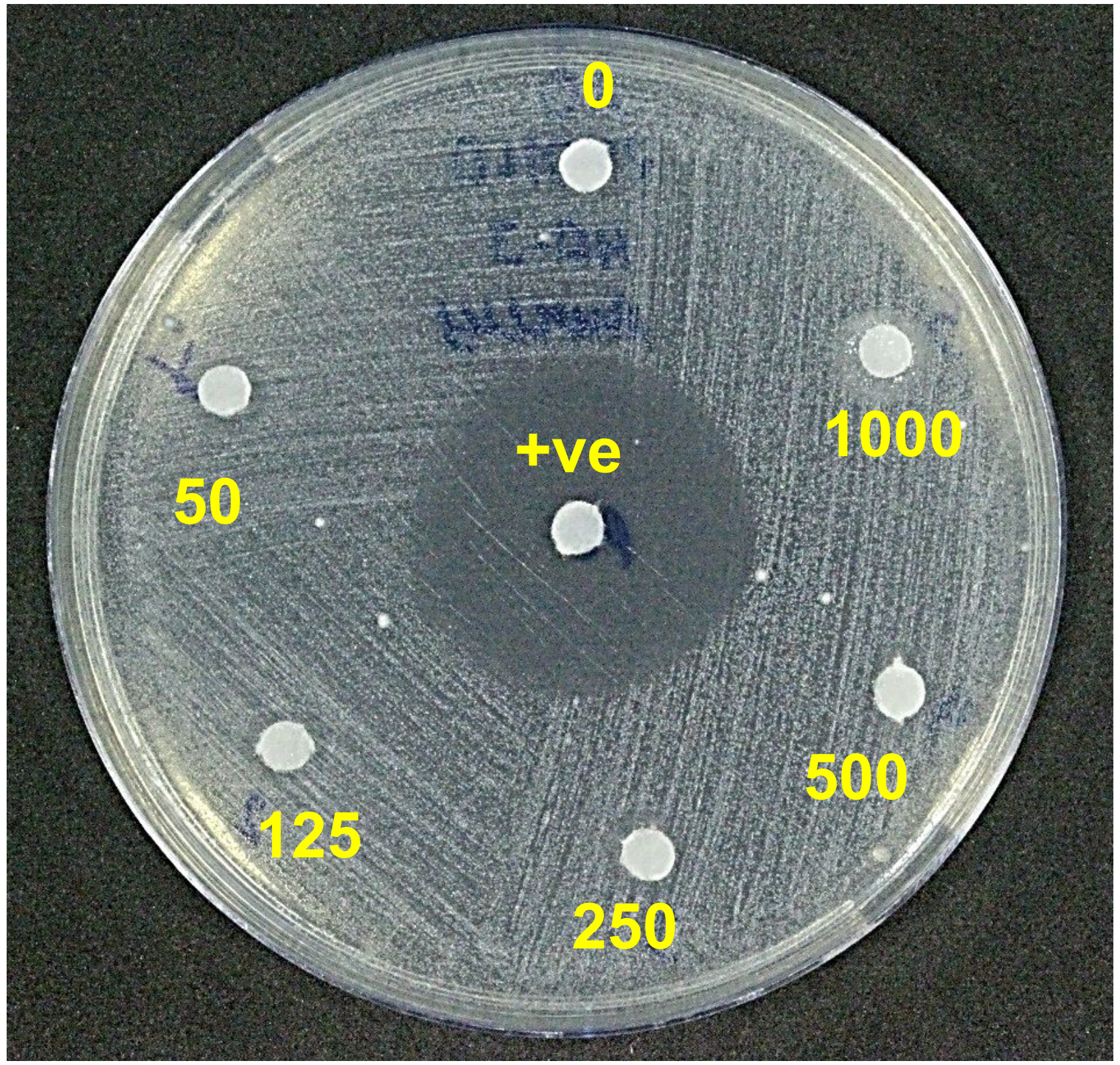

A

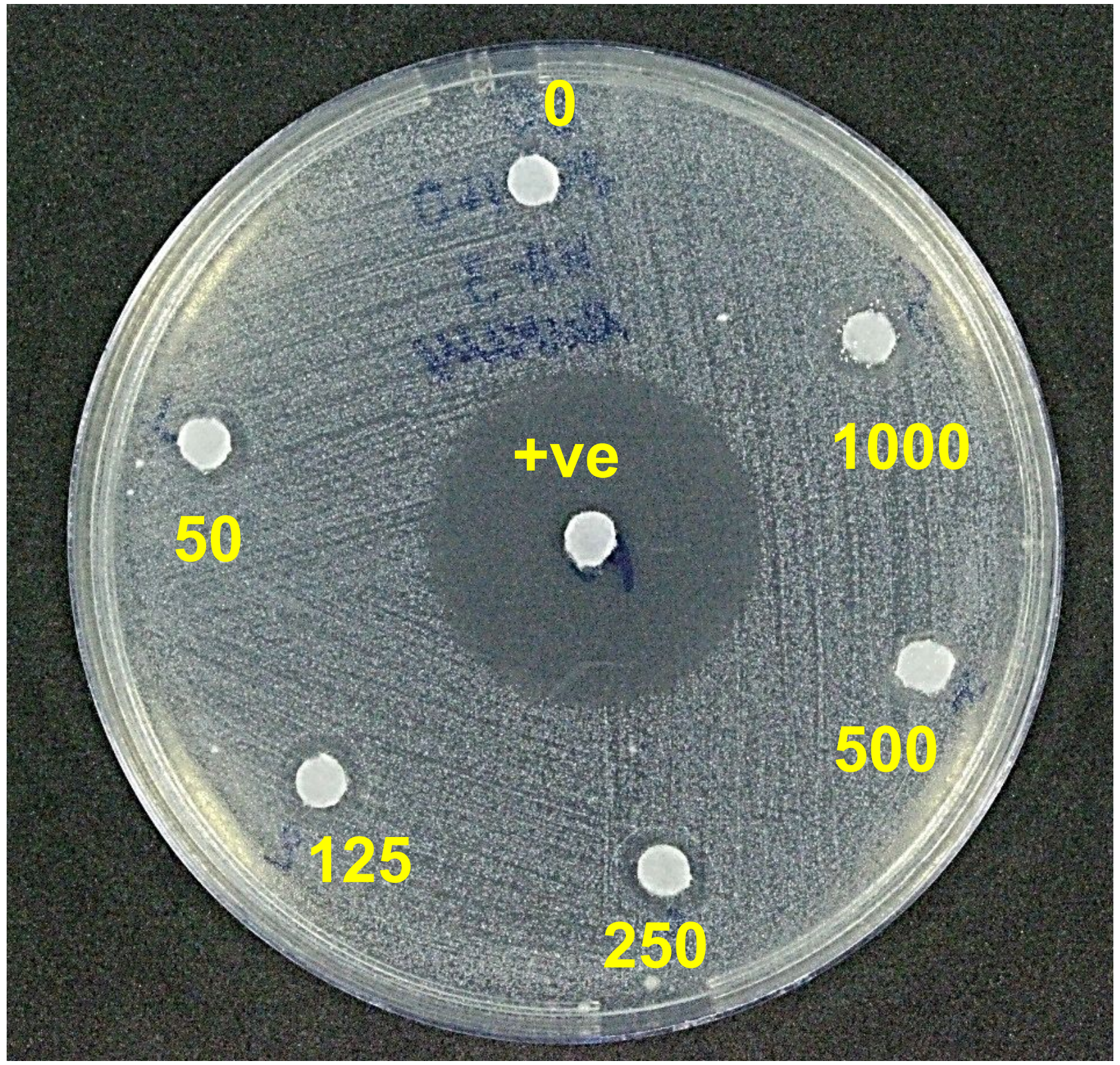

B

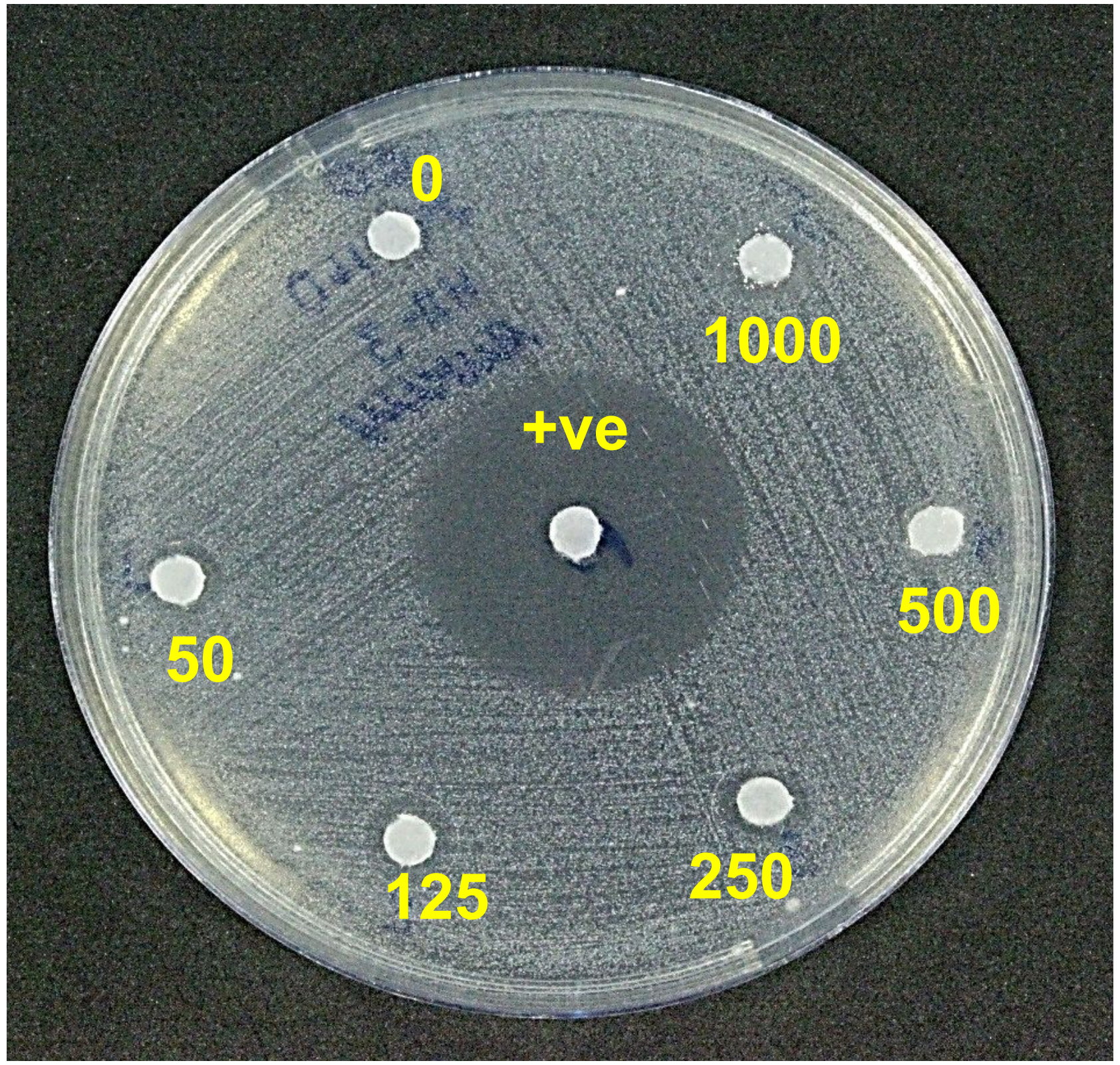

C

Amount present per disc in µg
Dispensed Volume - 10 µl
Positive Control (Ciprofloxacin) - 10 µg
